# Revealing topology with transformation optics

**DOI:** 10.1038/s41467-021-27008-x

**Published:** 2021-11-25

**Authors:** Lizhen Lu, Kun Ding, Emanuele Galiffi, Xikui Ma, Tianyu Dong, J. B. Pendry

**Affiliations:** 1 State Key Laboratory of Electrical Insulation and Power Equipment, School of Electrical Engineering, Xi’an Jiaotong University, 710049 Xi’an, China; 2 The Blackett Laboratory, Department of Physics, Imperial College London, London, SW7 2AZ UK; 3 Department of Physics, State Key Laboratory of Surface Physics, and Key Laboratory of Micro and Nano Photonic Structures (Ministry of Education), Fudan University, 200438 Shanghai, China; 4 Photonics Initiative, Advanced Science Research Center at the Graduate Center of the City University of New York, 85 St. Nicholas Terrace, New York, NY 10031 USA

**Keywords:** Photonic crystals, Nanophotonics and plasmonics, Transformation optics

## Abstract

Symmetry deepens our insight into a physical system and its interplay with topology enables the discovery of topological phases. Symmetry analysis is conventionally performed either in the physical space of interest, or in the corresponding reciprocal space. Here we borrow the concept of virtual space from transformation optics to demonstrate how a certain class of symmetries can be visualised in a transformed, spectrally related coordinate space, illuminating the underlying topological transitions. By projecting a plasmonic system in a higher-dimensional virtual space onto a lower-dimensional system in real space, we show how transformation optics allows us to construct a topologically non-trivial system by inspecting its modes in the virtual space. Interestingly, we find that the topological invariant can be controlled via the singularities in the conformal mapping, enabling the intuitive engineering of edge states. The confluence of transformation optics and topology here can be generalized to other wave realms beyond photonics.

## Abstract (web summary)

Visualizing and studying topological features is becoming crucial to develop new generation topological systems. Here the authors provide the theoretical and numerical demonstration of the potential of transformation optics connecting the virtual space to the physical space of lower dimension via conformal mapping.

## Introduction

Since its first deployment for the analytic design of electromagnetic cloaks, transformation optics (TO) has proven a powerful tool for both tailoring and understanding the physics of complex photonic structures^1–4^. In the nano-scale, insight into the response of complex plasmonic systems can be obtained by studying simpler, but spectrally equivalent geometries, due to the invariance of the in-plane scalar potential under a conformal mapping^5^. By leveraging TO, hidden symmetries in plasmonic systems can be revealed, empowering our physical intuition in understanding the flow of light in highly non-trivial nano-structures^6–9^. Moreover, TO enables the analytic design of hidden dimensions in electromagnetic systems, tailoring nano-structures to exhibit dramatic spectral effects such as ultra-broadband absorption^10,11^. Key to such exotic wave behavior is the geometrical singularities and hidden symmetries that can be built into a given coordinate transformation.

More recently the blossoming of the field of topological photonics enabled rapid developments in the design of devices such as photonic crystal cavities and optical isolators, as well as opening new directions such as topological lasing and photonic-qubit protection^12–17^. Topology describes a quantified global behavior of eigenmodes, which can be characterized by a topological invariant^18–23^. The early efforts focused on periodic structures, whereby topological invariants are defined on the band structure of a bulk crystal. However, it has been shown recently that symmetry indicators in real space provide an equivalent and elegant way to investigate topological materials^24–27^. Being rooted in symmetry, this approach is not limited to bulk bands of crystals. Therefore, a myriad of efforts has been devoted to the investigation of the topological interface, corner, and disclination modes in non-periodic systems, such as amorphous materials and quasi-crystals^28–36^. These topological phases have been characterized by Wannier functions, which constitute elementary basis states in real space. In many scenarios, however, the symmetry underlying a structure, which may be associated with a topological phase, is not directly evident in real space.

In this work, by exploiting the capability of TO to encode and decode a hidden symmetry, we demonstrate a scheme to probe the band topology from a virtual space, in which a spectrally related structure can provide intuitive physical insight into the band topology of the original system. Starting from a plasmonic system featuring a hidden, non-trivial symmetry, we design a conformal mapping with multiple singularities to construct a correlated plasmonic system in a virtual space, which can be projected onto a topologically non-trivial system with a lower dimensionality. Crucially, such singularities can be utilized to control the topological invariant for different bands, guiding the tailoring of edge states.

## Results

### Virtual space and hidden symmetry

**Fig. 1:**
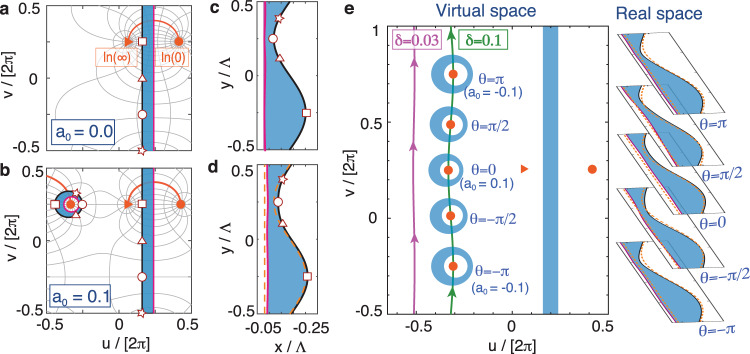
The multiplicity of a conformal mapping. Metasurface geometries in **a**, **b** the virtual space (u-v frame) and in **c**, **d** the real space (*x*–*y* frame). The relation between these two spaces is governed by the transformation in Eq. (1), and its contour plot containing the isolines of real and imaginary parts of *z* is shown by the light-gray solid lines in **a**, **b**. The filled orange circles and triangle markers in **a**, **b**, and **e** denote the ${{{{{{{\rm{ln}}}}}}}}(0)$ and ${{{{{{{\rm{ln}}}}}}}}(\infty )$ singularities of the mapping, respectively. The solid orange lines in **a** and **b** denote the branch cut of the mapping, terminating at the ${{{{{{{\rm{ln}}}}}}}}(0)$ and ${{{{{{{\rm{ln}}}}}}}}(\infty )$ singularities. All the magenta and black solid lines in **a**, **b** correspond to the boundary of the metasurface with the same colors shown in **c**, **d**, showing the multiplicity of the mapping. To further demonstrate such multiplicity, four points along the black line of the slab in **a**, **b** with coordinates (*u*, *v*) = (1, −*π*), (1, −*π*/2), (1, 0), and (1, *π*/2) are highlighted by the open stars, circles, triangles, and squares, and their equivalent points are shown by the same markers in **c**, **d** and in the eccentric cylinder of the virtual space in **b**. The values of *a*_0_ are 0 in **a** and **c**, and 0.1 in **b** and **d**. Other geometric parameters are Λ = 30*π* nm, w_0_ = 1.5, *u*_0_ = 1, and *d* = 0.5. **e** The left panel illustrates the geometries of the plasmonic metasurfaces in the virtual space for a complex *a*_0_ = *δ*exp(i*θ*). The two solid lines show the trajectories of the ln(0) singularities as *θ* varies from −*π* to *π*, for two values of *δ* = 0.1 (green) and *δ* = 0.03 (purple). Along the solid green line, we depict the eccentric cylinders with different values of *θ*, and their corresponding metasurfaces in the real space are shown in the right panel. The orange dashed lines highlight the metasurfaces when *δ* = 0.

We start with a class of plasmonic structures possessing a symmetry hidden in the virtual space^6,8^. Assuming that these plasmonic structures are deeply sub-wavelength in size, we denote them as metasurfaces. These metasurfaces (blue region) in Fig. 1c are constructed from a translationally invariant slab located between *u* = *u*_0_ and *u* = *u*_0_ + *d* (blue region) in Fig. 1a via the conformal mapping $z={{\Lambda }}/(2\pi ){{{{{{{\rm{ln}}}}}}}}\left(\frac{1}{{\rm e}^{w}-{{{{\rm i}}}}{{{{\rm w}}}}_{0}}+{{{{\rm i}}}}{\rm y}_{0}\right)$. Here, the complex *z* plane (*z* = *x* + *iy*) refers to the real space, while the *w* plane (*w* = *u* + *iv*) defines the virtual space. Λ is the period of the metasurface, ${{{{\rm w}}}}_{0}/{e}^{{u}_{0}}$ determines the modulation depth of the corrugated side of the metasurface (in black), and ${y}_{0}={{{{\rm w}}}}_{0}/({e}^{2({u}_{0}+d)}-{{{\rm w}}}_{0}^{2})$ ensures that the back-side (in magenta) remains flat upon the mapping (see Supplementary Note 1A for details).

**Fig. 2:**
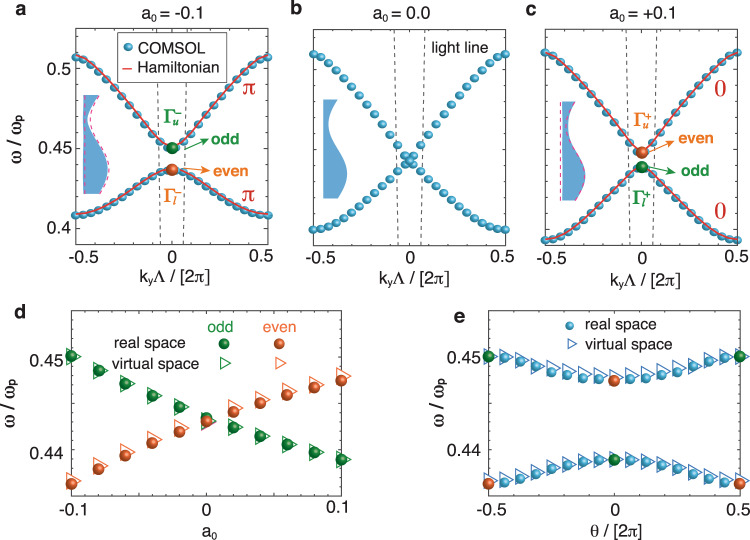
Band structures of the metasurfaces. **a**–**c** The band structures of the plasmonic systems with *a*_0_ = −0.1, 0, and +0.1, respectively. The states at the Γ-point in **a** and **c** are denoted by ${{{\Gamma }}}_{u}^{-}$, ${{{\Gamma }}}_{l}^{-}$, ${{{\Gamma }}}_{u}^{+}$, and ${{{\Gamma }}}_{l}^{+}$, in which the superscript stands for the sign of *a*_0_, and the subscript *u* and *l* denotes the upper and lower band. The gray dashed lines denote the light line, and the red solid lines are calculated via the Hamiltonian model. The insets in **a**–**c** show the schematics of the respective plasmonic metasurfaces, where the dashed lines highlight the metasurface boundary when *a*_0_ = 0. The material of the metasurfaces is described by a lossless Drude model with plasma frequency *ω*_*p*_ = 2 eV, while the background medium is vacuum. All other geometric parameters are the same as Fig. 1. **d** Eigenfrequencies at the Γ-point calculated in real space (filled circles) and in the virtual space (triangles) with *a*_0_ changing from −0.1 to +0.1. The color of the markers denotes the symmetry of the eigenstates, i.e., orange (green) represents the even (odd) mode. **e** The Γ-point eigenfrequencies calculated in the virtual space (triangles) and the real space (filled circles) with *δ* = 0.1 as a function of *θ*, showing that the bandgap is always open in this case. All the calculations are for transverse magnetic modes (*H*_*z*_ polarization).

From a conventional group symmetry point of view, the metasurface in Fig. 1c cannot have any symmetry-protected degeneracy. However, the TO-inspired virtual space makes the two-fold degeneracy at the Brillouin zone (BZ) center possible, which is also confirmed by the calculated band structures in Fig. 2b (see Supplementary Note 2A for details). Such two-fold degeneracy arises because transformation optics can relate the plasmonic modes of these metasurfaces to those of a uniform slab with band-folding-induced degeneracy in the virtual space at the BZ center. The conformal mapping preserves the in-plane permittivity and thus the magnetic fields under consideration. Such invariance endows the virtual space with an elegant and unique viewpoint to investigate the plasmonic bands. The real-space eigenmode solutions of the magnetic field shall have the form ${H}_{z}={e}^{i{k}_{y}y}{u}_{n,{k}_{y}}\left(x,y\right)$, with *n* and *k*_*y*_ being the band index and the Bloch wave vector, respectively. Transformation of *H*_*z*_ into the virtual space must bring about discontinuities due to the phase factor ${e}^{i{k}_{y}y}$. The periodic boundaries at *y* = ±0.5Λ in Fig. 1c are mapped to the branch cuts shown by solid orange lines in Fig. 1a, and thus the discontinuity in the virtual space happens when *H*_*z*_ crosses the branch cut (see Supplementary Note 1B for details). This indicates that the backscattering of plasmons in the real space effectively turns into the virtual-space discontinuity across the branch cut. When *k*_*y*_ = 0 (Γ point), the vanishing of the phase factor lets the discontinuity disappear, and so does the backscattering. This naturally leads to the two-fold degeneracy, which manifests itself by the band-folding feature of the slab dispersion from the virtual space (see Supplementary Note 2B for details).

### Multi-valued mapping and topological transitions

By noting that the closing of a bandgap always empowers us to realize topological transitions, it is natural to ask whether the singularities of a conformal mapping can serve as a tool to design topological phases. The ${{{{{{{\rm{ln}}}}}}}}$(∞) and ${{{{{{{\rm{ln}}}}}}}}(0)$ singularities in Fig. 1a correspond to +*∞* and −*∞*, respectively, in real space. Thus, the ${{{{{{{\rm{ln}}}}}}}}$(∞) singularity can be treated as the electromagnetic source in the virtual space, since the electromagnetic waves are incident from the right-hand side in the real space, while the ${{{{{{{\rm{ln}}}}}}}}(0)$ singularity is related to the shape of the metasurface. Importantly, the aforementioned conformal mapping is a one-to-one mapping, and thus the eigenmodes are also in a one-to-one correspondence between these two spaces, indicating a limitation in the number of degrees of freedom available to manipulate the eigenmodes. To conquer this limitation, we introduce an extra term in the conformal mapping as

1 $$z=\frac{{{\Lambda }}}{2\pi }{{{{{\rm{ln}}}}}}\left[\frac{1}{{{{{{\rm e}}}}}^{w}-{{{{\rm i}}}}{{{{{\rm w}}}}}_{0}}+{{{{{\rm iy}}}}}_{0}+{a}_{0}{{{{{\rm e}}}}}^{-w}\right],$$

where *a*_0_ = *δe*^*iθ*^ is a complex number. This transformation involves two pairs of ${{{{{{\mathrm{ln}}}}}}}\,(0)/{{{{{{\mathrm{ln}}}}}}}$ (∞) singularities due to the presence of the term *a*_0_*e*^−*w*^ (see Supplementary Note 1 for details). For demonstration purposes, Fig. 1b shows the *a*_0_ = 0.1 case, where we see that not only the original slab but also an eccentric hollow cylinder in the virtual space are now mapped into the same plasmonic metasurface (Fig. 1d) by the transformation, as a result of the two-fold multiplicity of the mapping. Figure 1b also shows that the position of the cylinder corresponds to another ${{{{{{{\rm{ln}}}}}}}}(0)$ singularity. Mathematically speaking, this multiplicity results from the two solutions of the inverse transform of Eq. (1), which form two distinct images in the virtual space from a single metasurface in real space. As we set out to demonstrate, this multiplicity offers us the opportunity to investigate topological transitions in the virtual space.

We further show in Fig. 1e how the geometries vary when *a*_0_ is complex. The amplitude *δ* affects the separation between the slab and the cylinder, *i.e*. smaller values of *δ* result in a larger distance, such that when *a*_0_ → 0 the cylinder shifts towards infinity in the virtual space, and the system reverts to the one shown in Fig. 1a. Meanwhile, the variation of *θ* from −*π* to *π* results in a smooth translation of the ${{{{{{{\rm{ln}}}}}}}}(0)$ singularity from *v* = −0.25*π* to *v* = 0.75*π*. It is crucial to notice that despite the dramatic changes of the geometry in the virtual space, the extra term *a*_0_*e*^−*w*^ bears only a perturbative effect on the geometry of the resulting metasurfaces in the real space, shown in the right panel of Fig. 1e. However, as we set out to show, the structural changes in the virtual space witness the underlying occurrence of a topological transition.

To verify the occurrence of a topological transition, we show in Fig. 2a–c the calculated band structures of the metasurfaces for *a*_0_ = −0.1, 0, and +0.1, respectively. The two-fold degeneracy is lifted, and a gap opens at the Γ-point. When *a*_0_ is a real number, i.e. for *θ* = 0, ±*π*, the resulting metasurfaces always possess mirror symmetry, as shown in the inset of Fig. 2a, c. Tracking the symmetry of the eigenmodes as *a*_0_ varies from −0.1 to +0.1 clearly reveals that even and odd modes undergo a crossing at the Γ-point, implying that not only the location of the ${{{{{{\mathrm{ln}}}}}}}\,(0)$ singularity breaks the two-fold degeneracy, but the sign of *a*_0_ is in fact responsible for a band inversion. Thus, the metasurfaces experience a topological transition, shown explicitly by the filled circles in Fig. 2d. To quantify this topological transition, we have also constructed a two-band Hamiltonian model based on the shape deformation of plasmonic metasurfaces^37,38^ (Supplementary Note 3), and the calculated bands shown as red lines in Fig. 2a, c agree well with the fully numerical results (blue dots). The calculated Zak phases of each band are indicated in Figs. 2a, c, thereby confirming the topological transition.

Transitions between symmetry-protected topological phases may be induced either by closing the gap (e.g., through *a*_0_ = 0, see Fig. 2d) or by breaking the relevant symmetry. In Fig. 1e, we show how a complex value of *a*_0_ breaks mirror symmetry in real space, such that the topological transition between the *a*_0_ = −0.1 and +0.1 scenarios can be smooth, with the gap always open, as shown by the filled circles in Fig. 2e. In what follows, we propose an alternative and elegant way based on TO to shed light on the band inversion, while circumventing the need for any computational effort.

### The virtual space representation

**Fig. 3:**
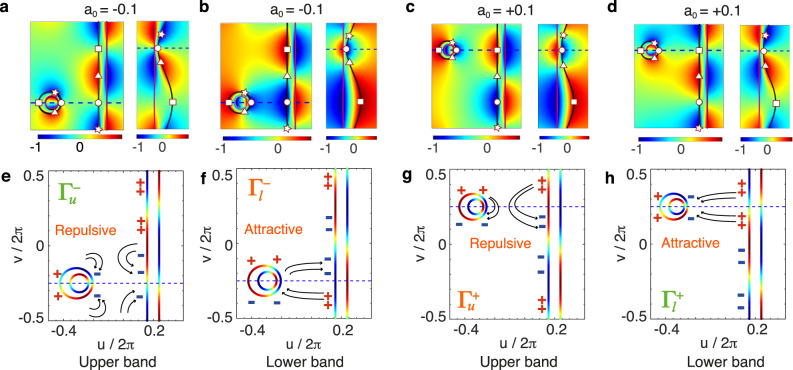
Projection of the eigenstates and schematic TO illustration of the topological transition. **a**–**d** The calculated Im(*H*_*z*_) distributions for the four eigenstates marked by ${{{\Gamma }}}_{u}^{-}$, ${{{\Gamma }}}_{u}^{+}$, ${{{\Gamma }}}_{l}^{-}$, and ${{{\Gamma }}}_{l}^{+}$ in Fig. 2 in the virtual space (left color plots) and in the real space (right color plots). The star, circle, triangle, and square markers herein share the same notation with Fig. 1, which helps us understand the projection of the eigenstates. **e**–**h** The schematics of the magnetic fields and electric charges in the virtual space. The color plots at the boundaries stand for Im(*H*_*z*_), which ranges from −1 to 1, while the arrow lines and the ± symbols denote the electric field lines and the sign of the related surface charge distributions, respectively, from which the interaction between the cylinder and the slab can be characterized as repulsive or attractive. The dashed lines in **a**–**h** show the symmetry plane in two spaces.

We are now ready to show how the band inversion can be characterized by examining the coupled slab-cylinder system in the virtual space, thus providing a framework to predict the band topology by simply leveraging the mode profiles of a slab. We first calculate the band structures of the virtual-space coupled system, and the Γ-point eigenfrequencies are shown by the open triangles in Fig. 2d, e, which are consistent with the corresponding eigenfrequencies in real space (see Supplementary Note 4 for details). As opposed to the one-to-one mapping case, the multi-valued nature of the mapping implies that each eigenfrequency of the coupled system in the virtual space is associated to a pair of two-fold degenerate eigenstates. However, it can be shown that only one of these degenerate states is physical, as the consistency of the electromagnetic fields between the slab, the cylinder and the metasurface must be preserved upon the mapping (see Supplementary Note 4 for details). Figure 3a–d shows the Im(*H*_*z*_) distributions of the eigenstates in the real space (right plots) and the TO-allowed eigenstates in the virtual space (left plots), respectively, for *a*_0_ = −0.1 (Fig. 3a, b) and *a*_0_ = +0.1 (Fig. 3c, d). Such a TO-allowed eigenstate is a linear combination of the two-fold degenerate states. It can be inferred that the dimensionality of the Hilbert space in the virtual space is doubled by the multi-valued mapping, and the eigenstates in the real space constitute a lower-dimensional projection of the virtual space.

The oscillating feature of Im(*H*_*z*_) along the slab boundaries shown in Fig. 3a–d implies that the cylinder in the virtual space can be treated as a perturbation to the system with a single slab. This allows us to deploy the known mode profiles of a single slab to investigate the topological transition without performing any rigorous calculation, but simply inspecting the geometric effect of the conformal map. The projection of the states restricts the field profiles and the signs of the charge at the cylinder surface relative to the slab surface, as shown schematically in Fig. 3e–h. Let us start from the eigenmode of the slab in which the field distributions at *u* = *u*_0_ is described by $\cos (v)$. For real *a*_0_ < 0 [Fig. 3e], the interaction between the cylinder and the slab is repulsive, as charges of the same sign face each other. This in turn implies that this mode has a higher frequency. As *a*_0_ flips from negative to positive values, the effect of the transformation is to push the location of the cylinder to *u* → −*∞* and back to the original out-of-plane position, but shifted along the in-plane axis *v* by half a unit cell [Fig. 3h]. According to the correlation between eigenstates of the slab and the cylinder, the interaction between the two structures is now attractive, since opposite charges face each other.

Remarkably, assuming the only prior knowledge of the multi-valued conformal map from which the non-trivial grating structure originates, and deploying only the trivial eigenmodes of a flat plasmonic slab in the virtual frame, the band inversion can be accurately predicted, including the exact critical point at which the transition occurs. The same argument can be applied to the eigenmode of the slab in which the field distributions at *u* = *u*_0_ is described by $\sin (v)$ [Fig. 3f, g], where the quadrupolar interaction with the corresponding charge distribution on the slab can be seen by inspection to be attractive for *a*_0_ < 0 [Fig. 3f] and repulsive for *a*_0_ > 0 [Fig. 3g]. Again, this is due to the shifting of the location of the cylinder by half a unit cell, which results from the sign flip of *a*_0_. We also give a proof of the band inversion with a standard Hamiltonian approach (see Supplementary Note 3 for details). Importantly, however, such arguments cannot be inferred directly in real space, where no physical insight can be drawn from the minor differences between the metasurfaces. Instead, with the help of TO, the band inversion in the real space can be understood directly as a simple mode-coupling between the two transformed plasmonic structures in the virtual space.

**Fig. 4:**
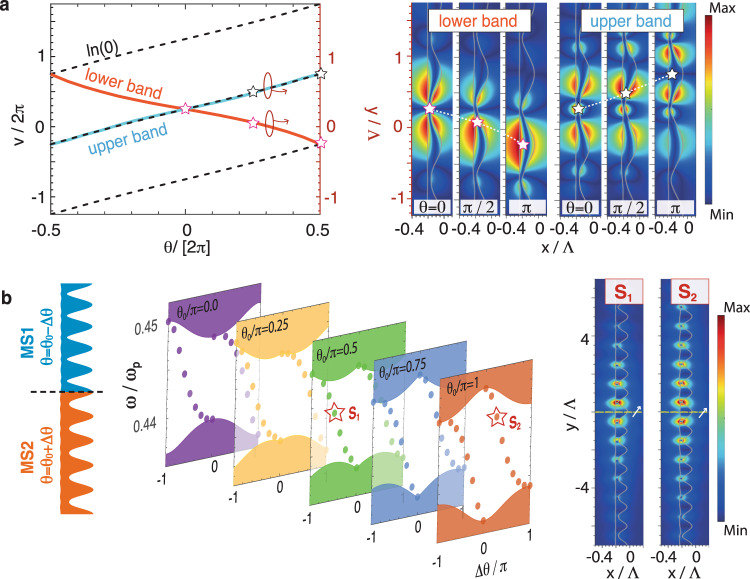
Wannier functions and edge modes. **a** The orange and cyan solid lines depict the *y* position (marked on the right vertical axis) of the Wannier function centers (WFCs) in the real space for the lower and upper band, respectively. Here, the symmetry center is chosen at *y* = 0.25Λ. For reference, the *v*-coordinates of the ${{{{{{{\rm{ln}}}}}}}}(0)$ singularities (the left vertical axis) as a function of *θ* are shown as dashed lines in **a**. Shown in the right panel are the Wannier functions ∣*H*_*z*_∣ of the lower (left panel) and upper (right panel) bands for *θ* = 0, *π*/2, and *π* (left to right for each panel). The corresponding WFCs are marked by the open stars. **b** Edge modes of the combined metasurface system. The left panel shows a schematic illustration of the edge, which is formed by two metasurfaces with different *a*_0_, corresponding to ${a}_{0}=0.1\exp [i({\theta }_{0}+{{\Delta }}\theta )]$ and ${a}_{0}=0.1\exp [i({\theta }_{0}-{{\Delta }}\theta )]$, respectively. The connected point is placed at *y* = 0. Shown in the middle are the edge modes as a function of Δ*θ*, for different values of *θ*_0_, each denoted by a different color. The shaded regions denote the bulk bands. The calculation is performed via the supercell approach, with 16 unit cells for each metasurface. The amplitudes of the electric field for the states *S*_1_ (Δ*θ* = −*π*/2 and *θ*_0_/*π* = 0.5) and *S*_2_ (Δ*θ* = +*π*/4 and *θ*_0_/*π* = 1) under an electric dipole excitation are shown in the right panel. Here, the dipole is oriented in the *x* − *y* plane, with a 45° angle with respect to the *y* axis.

The previous analysis shows that the position of the ${{{{{{{\rm{ln}}}}}}}}(0)$ singularity (the cylinder) is closely related to the topological transition. To further investigate the role of the singularities, we calculate the Wannier functions of these bands^39^. Wannier functions are defined as $\left|{{{{{{{\bf{R}}}}}}}}n\right\rangle =\frac{V}{{(2\pi )}^{3}}{\int}_{{{{{{{{\rm{BZ}}}}}}}}}d{{{{{{{\bf{k}}}}}}}}{e}^{-i{{{{{{{\bf{k}}}}}}}}\cdot {{{{{{{\bf{R}}}}}}}}}\left|{u}_{n{{{{{{{\bf{k}}}}}}}}}\right\rangle$, where *V* is the real-space primitive cell volume and $\left|{u}_{n{{{{{{{\bf{k}}}}}}}}}\right\rangle$ is the Bloch wavefunction after applying a smooth gauge (see Supplementary Note 5 for details)^40^. The location of the Wannier function centers (WFCs) is an unequivocal signature of a topological transition. Shown in Fig. 4a are the *y* positions of the WFCs for the upper and lower bands as *θ* varies from −*π* to *π*. We notice a smooth one-lattice shift of the WFCs of both bands as *θ* ranges from −*π* to *π*. In order to visualize the relation between the WFCs and the singularities of the conformal map, we plot in Fig. 4a the evolution of the ${{{{{{{\rm{ln}}}}}}}}(0)$ singularity in the *v*-direction (black dashed lines), indicating that the WFCs (light blue and red curves for the upper and lower band, respectively) can be controlled via the singularity. The amplitude ∣*H*_*z*_∣ of the Wannier functions is shown in the right panel of Fig. 4a, highlighting how the trajectories of the WFCs for the upper band and the lower band shift in opposite directions as *θ* increases from 0 to *π*. It should be noted that the shift of WFCs in the *y* direction must be an integer multiple of the lattice constant, which depends on the band index. This indicates that the proposed framework can be used to determine the topological phases of different bands over the entire BZ of the plasmonic system (see Supplementary Note 6 for details).

### Edge states

Topologically non-trivial systems exhibit a bulk-boundary correspondence, and the position of the WFCs predicts whether or not a band gap will host edge modes^41^. In Fig. 4b, we construct a domain wall formed by two metasurfaces with different *a*_0_. The frequencies of the edge mode inside the gap for different values of *θ*_0_ are shown in the center panel of Fig. 4b, where we plot the projected eigenmodes as a function of Δ*θ*. For each series of metasurfaces with the same *θ*_0_, the continuous shifting of the edge state from the upper bulk band to the lower one with Δ*θ* changing from −*π* (0) to 0 (*π*) inside the bandgap further confirms the topological transition. Finally, in the right panel of Fig. 4b, we probe two edge modes (labeled *S*_1_ and *S*_2_ in the middle panel) by placing an electric dipole on top of the domain wall. The electric field distributions of the edge states *S*_1_ and *S*_2_ are both localized at the interface of the combined system, demonstrating the excitation of the edge states. Such TO-based edge states reveal that the symmetry hidden in the virtual space provides another class of symmetry to harness in topology, and a very recent fabrication technique fulfills the requirement of high spatial resolution needed for the realization of our proposed topological metasurfaces^42^. From a design point of view, the merits of our method are two-fold. On the one hand, the working frequency can be determined simply by the dispersion of a slab. On the other hand, whether the system is topologically trivial or not can be intuitively characterized from the mode-coupling in the virtual space.

## Discussion

In this work, we propose the analytical formalism of TO to provide an intuitive framework for understanding the band topology of a complex plasmonic system, by mapping it to a spectrally related system in a virtual space, where the eigenstates in the real space constitute a lower-dimensional projection of the virtual space. Importantly, we unveil that the topological properties of a plasmonic system can be traced back to the singularities in the conformal map used to generate a structure. As here we only deal with the ${{{{{{{\rm{ln}}}}}}}}(0)$ singularity, other types of singularities can also be leveraged in the virtual space for the study of topological matter. Since we here only investigate the square-root singularity, it is natural to ask how the topological properties of a system could be tuned with a conformal mapping involving higher-order singularities, such as a cubic-root singularity.

The conformal mapping here only concerns a 1D translationally invariant structure, but the conformal mapping can be more general. For example, similar to the slab and metasurface case, an ellipse and an annulus also share the same eigenstates, and thus we can define a so-called hidden rotational symmetry^5^. Combining this with lattice symmetry offers an extra degree of freedom to control the topological bands of 2D and 3D photonic crystals. This framework, therefore, opens an alternative perspective for the study of higher-order topological features such as corner states^43,44^. Finally, the proposed strategy may be extended to the fields of acoustics, elasticity, and spintronics, thus providing unprecedented physical insight for the analytical design of topologically non-trivial structures.

## Supplementary information

Author Checklist

Supplementary Information

## Data Availability

The data that support the plots within this paper and other findings of this study are available from the corresponding author upon reasonable request.

## Code availability

The computer codes that support the plots within this paper and the findings of this study are available from the corresponding author upon reasonable request.
