# Supplementary material for: Revealing topology with transformation optics
Source: Nat Commun. 2021 Nov 25;12:6887. doi: 10.1038/s41467-021-27008-x (PMC8617177; doi:10.1038/s41467-021-27008-x)
Supplement: Supplementary file 2 — Supplementary Information [file 41467_2021_27008_MOESM2_ESM.pdf]

Supplementary Information: Revealing Topology with Transformation Optics

Lizhen Lu,[1,2] Kun Ding,[2,3,*] Emanuele Galiffi,[2,4] Xikui Ma,[1] Tianyu Dong,[1] and J. B. Pendry[2,†]

[1] *State Key Laboratory of Electrical Insulation and Power Equipment, School of Electrical Engineering, Xi'an Jiaotong University, Xi'an 710049, China*

[2] *The Blackett Laboratory, Department of Physics, Imperial College London, London SW7 2AZ, United Kingdom*

[3] *Department of Physics, State Key Laboratory of Surface Physics, and Key Laboratory of Micro and Nano Photonic Structures (Ministry of Education), Fudan University, Shanghai 200438, China*

[4] *Photonics Initiative, Advanced Science Research Center at the Graduate Center of the City University of New York, 85 St. Nicholas Terrace, 10031 New York, NY, United States*

CONTENTS

Supplementary Note 1. The mapping of the coordinates and the electromagnetic fields 1
A. Mathematical details of conformal mapping 2
B. Field discontinuities across the branch cuts 3

Supplementary Note 2. Band structures and the conformal symmetry 4
A. Calculation of the Band Structures 4
B. Conformal Symmetry 5

Supplementary Note 3. A Two-band Hamiltonian Model 5
A. Definition and Notations 6
B. Formulation of the Hamiltonian 7
C. Understanding the band inversion from the Hamiltonian approach 8
D. Orthogonality of the eigenstates 11

Supplementary Note 4. Correlation of the eigenstates between two spaces 13
A. The band structures of the plasmonic system in the virtual space 13
B. Projection of the eigenstates 13

Supplementary Note 5. Calculation of the Wannier functions 15

Supplementary Note 6. Discussion on the higher order bands 15

References 17

Supplementary Note 1. THE MAPPING OF THE COORDINATES AND THE ELECTROMAGNETIC FIELDS

As the core ingredient, the multi-valued mapping transforms both the geometry and the electromagnetic fields from the virtual space to the real space and vice versa. Therefore, we show the details of geometric parts and electromagnetic fields in Supplementary Note 1.A and Supplementary Note 1.B, respectively.

* kunding@fudan.edu.cn
† j.pendry@imperial.ac.uk

A. Mathematical details of conformal mapping

In this section, we show the singularities and the branch cuts of the transformation. The conformal mapping is described by

$$z = \frac{\Lambda}{2\pi} \ln\left(\frac{1}{e^{w} - \mathrm{iw}_0} + \mathrm{iy}_0 + \mathrm{a}_0\mathrm{e}^{-w}\right). \tag{S1}$$

When $a_0 = 0$, the inverse transform has the same functional form, and is given by

$$w = \ln\left(\frac{1}{e^{2\pi z/\Lambda} - \mathrm{i}y_0} + \mathrm{iw}_0\right), \tag{S2}$$

When a non-zero a_0 is imposed, there are two solutions to the inversion transform

$$w_1 = \ln\left(\frac{\mathrm{i}(1 + a_0 + \mathrm{i}e^{2\pi z/\Lambda}\mathrm{w}_0 + \mathrm{w}_0 y_0 + \sqrt{-4\mathrm{i}a_0\mathrm{w}_0(e^{2\pi z/\Lambda} - \mathrm{i}y_0) + (1 + a_0 + \mathrm{i}e^{2\pi z/\Lambda}\mathrm{w}_0 + \mathrm{w}_0 y_0)^2})}{2(\mathrm{i}e^{2\pi z/\Lambda} + y_0)}\right), \tag{S3}$$

and

$$w_2 = \ln\left(\frac{\mathrm{i}(1 + a_0 + \mathrm{i}e^{2\pi z/\Lambda}\mathrm{w}_0 + \mathrm{w}_0 y_0 - \sqrt{-4\mathrm{i}a_0\mathrm{w}_0(e^{2\pi z/\Lambda} - \mathrm{i}y_0) + (1 + a_0 + \mathrm{i}e^{2\pi z/\Lambda}\mathrm{w}_0 + \mathrm{w}_0 y_0)^2})}{2(ie^{2\pi z/\Lambda} + y_0)}\right). \tag{S4}$$

The complex logarithm has singularities at the origin and infinity, respectively. The singularities of Eq. S1 with $a_0 = 0$ are

- $\ln(\infty)$

$$w = \ln(\mathrm{iw}_0) + 2n\pi i \tag{S5}$$

- $\ln(0)$

$$w = \ln\left[\frac{i(1 + \mathrm{w}_0 y_0)}{y_0}\right] + 2n\pi i = 2(u_0 + d) + \pi - \ln(i\mathrm{w}_0) + 2n\pi i \tag{S6}$$

When a_0 is non-zero, there are two pairs of $\ln(0)/\ln(\infty)$ singularities, which are

- $\ln(\infty)$

$$w_{\infty,1} = \ln(\mathrm{iw}_0) + 2n\pi i \tag{S7}$$

$$w_{\infty,2} = -\infty \tag{S8}$$

- $\ln(0)$

$$w_{0,1} = \ln\left(\frac{\mathrm{i}(1 + a_0 + \mathrm{w}_0 y_0 - \sqrt{-4a_0\mathrm{w}_0 y_0 + (1 + a_0 + \mathrm{w}_0 y_0)^2})}{2y_0}\right) + 2n\pi i \tag{S9}$$

$$w_{0,2} = \ln\left(\frac{\mathrm{i}(1 + a_0 + \mathrm{w}_0 y_0 + \sqrt{-4a_0\mathrm{w}_0 y_0 + (1 + a_0 + \mathrm{w}_0 y_0)^2})}{2y_0}\right) + 2n\pi i \tag{S10}$$

Here, n refers to an arbitrary integer, therefore the singularities in the virtual space are an array of branch points, which correspond to the $-\infty(+\infty)$ in the real space. The left-hand side of Supplementary Fig. 1 shows a typical distribution of the $\ln(0)/\ln(\infty)$ singularities and the branch cuts in the virtual space for a particular n. The periodic boundaries at $y = \pm 0.5\Lambda$ shown by the solid orange line in the right-hand side of Supplementary Fig. 1 are mapped to the two branch cuts in the virtual space simultaneously. This reflects the multiplicity of the conformal mapping defined in Eq. (S1).

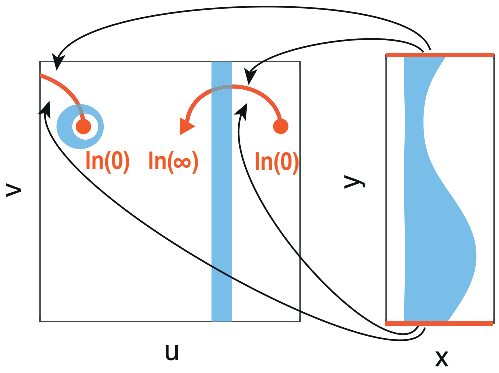

Supplementary Fig. 1. Two logarithm singularity pairs in the virtual space and their mapping in the real space. The filled triangle, circles, and solid lines correspond to the $\ln(\infty)$ singularity, the $\ln(0)$ singularity, and branch cuts, respectively.

B. Field discontinuities across the branch cuts

Since we focus on plasmonics for the transverse magnetic (TM) polarization case here, there are no in-plane components of the magnetic field, namely only z components leave. The conformal mapping then guarantees that the magnetic field is invariant in two spaces under electrostatic limit, and thus the magnetic field distributions in the virtual space can be obtained by the coordinate transformation from the real space.

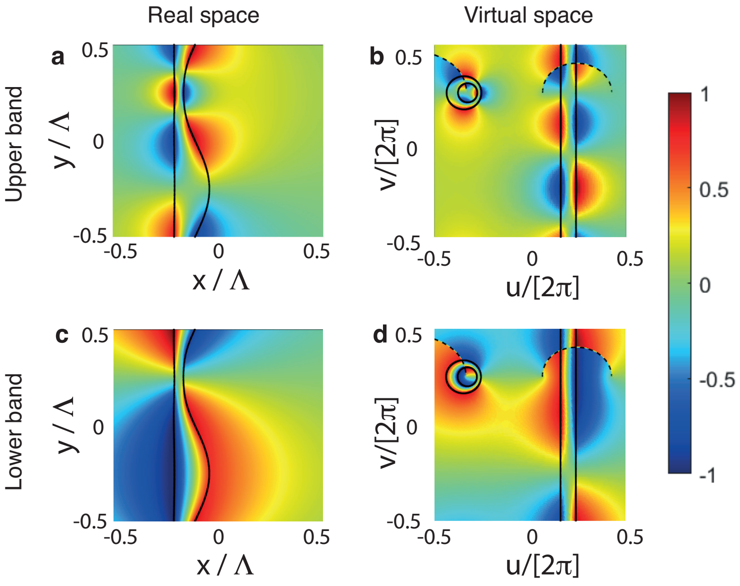

Supplementary Fig. 2. Imaginary parts of the magnetic field distributions in the real space with $a_0 = 0.1$ at X point for the upper band (a) and for the lower band (c). (b) and (d) show the virtual-space fields transformed from the corresponding eigenstates in (a) and (b), respectively. The dashed lines in (b) and (d) denote the branch cuts.

The magnetic fields in the real space satisfy $H_z^z(x,y) = e^{ik_y y} u_{n,k_y}(x,y)$ with n and k_y being the band index and the Bloch wave vector respectively. Here, $u_{n,k_y}(x,y) = u_{n,k_y}(x,y+\Lambda)$ is the Bloch wave function. When k_y is non-zero, $H_z^z(x,y)$ is discontinuous across the periodic boundaries, causing the discontinuity across the branch cuts in the virtual space. Shown in Supplementary Fig. 2 are the field distributions at X point in two spaces with $a_0 = 0.1$, implying such discontinuity. This indicates that the boundary conditions in two spaces are not equivalent when a finite k_y is imposed, and we can deal with the modes of the metasurfaces away from the zone center either via a quasistatic method or by solving the full set of Maxwell's equations[1, 2]. However, such discontinuity disappears when $k_y = 0$ (Γ point). Therefore, at the BZ center, the correlation between the eigenstates in two spaces can be constructed. In short, the singularities mainly determine the geometric shape, while the branch cuts relate to the correlation of eigenstates between real space and virtual space.

Supplementary Note 2. BAND STRUCTURES AND THE CONFORMAL SYMMETRY

A. Calculation of the Band Structures

The eigenvalue equation for our plasmonic system can be expressed using the electric field with the form

$$\nabla \times \left(\frac{1}{\mu}\nabla \times \boldsymbol{E}\right) - \epsilon\frac{\omega^2}{c^2}\boldsymbol{E} = 0. \tag{S11}$$

In our work, we deal with the TM polarization, in which $\boldsymbol{E} = (E_x, E_y)^T$. The permeability is $\mu = 1$, and the permittivity is described by the lossless Drude model with $\epsilon = 1 - \omega_p^2/\omega^2$. Then the partial differential equation of a dispersive photonic crystal is described by

$$\nabla \times \left(\frac{1}{\mu}\nabla \times \boldsymbol{E}\right) + \left(\frac{\omega_p}{c}\right)^2 \mathbf{E} = \left(\frac{\omega}{c}\right)^2 \mathbf{E}. \tag{S12}$$

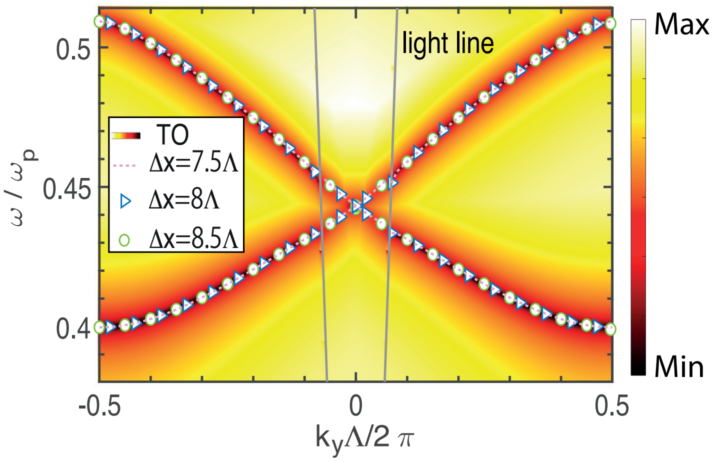

Supplementary Fig. 3. The band structures obtained from the eigenvalue solver and transformation optics approach. The color plot illustrates the poles of the reflection spectrum via TO; the dashed line, the triangle markers and the square markers denote the band structures obtained from the eigenvalue solver, with Δx being 7.5Λ, 8Λ, and 8.5Λ, respectively. Here, Δx is the period along x in the calculation. Other parameters are: a_0=0, $\omega_p = 2$ eV, $\Lambda = 30\pi$ nm, $w_0 = 1.5$, $u_0 = 1$, and $d = 0.5$.

By solving Eq. S12 using COMSOL, the corresponding eigenstates can be obtained. In the eigenvalue solver, the Bloch wave vector k_y is imposed to the system, and the periodic boundary condition is set to the boundaries along both x and y directions, satisfying the Bloch's theorem.

The periodic boundary condition is used in the x-direction numerically. We here deal with the plasmonic modes along y direction, and thus the simulation region in the x-direction must be chosen to get rid of the interaction between the plasmonic modes and the cavity modes to keep the physics we wish to demonstrate intact.

To show the band calculation is independent of the period Δx along the x direction in our simulation, we have firstly compared the calculated eigenstates with the TO-based analytic approach[3], which are in good agreement as shown in Supplementary Fig. 3. Furthermore, Supplementary Fig. 3 also shows the band structures for $a_0 = 0$, with Δx being 7.5Λ, 8Λ, and 8.5Λ. The good agreements indicate the choice of Δx does not alter the physics of interest.

B. Conformal Symmetry

Different from the Dirac points which results from the lattice symmetry and can be obtained from the character table, the two-fold degeneracy in this work is due to the equivalent eigenvalue solutions between the slab and the grating when the Bloch wave vector k_y vanishes. Therefore, the frequency of the topological transition point can be intuitively controlled in the virtual space from the dispersion of a slab.

As shown in Supplementary Fig. 4, a class of metasurfaces with different modulation depths can be designed from their 'mother structure', a plasmonic slab, from which their eigenmodes at the zone center are fixed, keeping consistent with this slab in the virtual space. This additional advantage over the lattice symmetry may be beneficial to the design of the edge state. By imposing a_0 in the conformal mapping, the gap opens where the center of the gap can be fixed at the band-crossing point (the yellow stars in Supplementary Fig. 4), indicating the frequency of the edge state can be designed by simply deploying a slab dispersion, without any real space calculation.

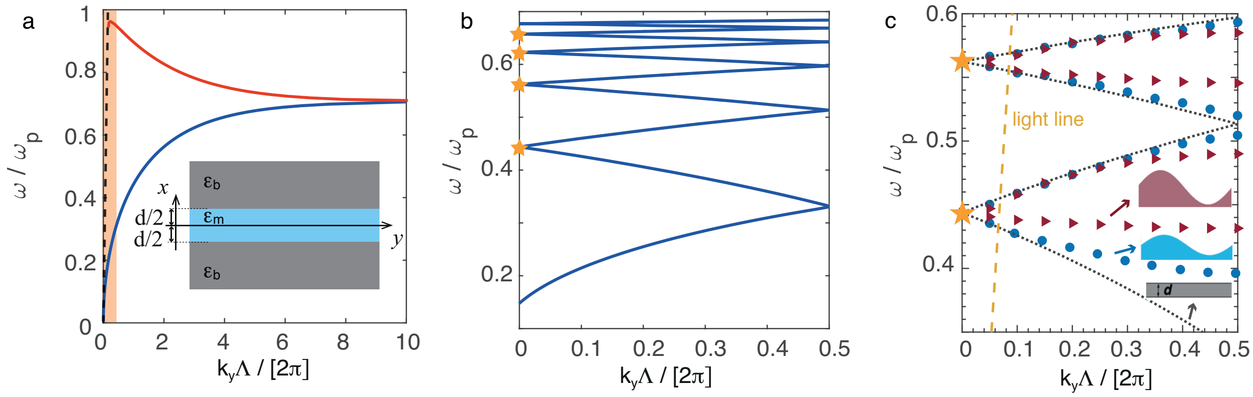

Supplementary Fig. 4. Schematic illustration of the conformal symmetry. (a) Dispersion of a plasmonic slab, and (b) its folded representation with an artificially imposed periodicity $\Lambda = 94.8$ nm. (c) Band structures for a class of conformal metasurfaces which are generated from the slab in the virtual space via conformal mapping are shown by the triangle and circle markers, and the folding of the slab dispersion is shown in the dotted line. The yellow stars in (b-c) denote the central frequency pinned down by the conformal symmetry.

Supplementary Note 3. A TWO-BAND HAMILTONIAN MODEL

In this section, a two-band Hamiltonian model is formulated, which helps us to understand the band inversion in the plasmonic system when a_0 is imposed, which constitutes a complementary part with the interpretation from the virtual space as discussed in the main text. The schematics of the problem is shown in Supplementary Fig. 5. A dispersive metasurface with the period Λ is deformed into a perturbed one with the boundary

changing from $\partial\Omega_u$ to $\partial\Omega_p$. The problem we set out to solve in this section is to establish the Hamiltonian dealing with the shape deformation of such a dispersive periodic structure. Inspired by a recent work which deals with the perturbation theory of dispersive nano-particle, we here deploy this method as the theoretical building block and extend it to deal with the band structures of the photonic crystals (Supplementary Note 3.A and 3.B) [4]. Applying such method to the plasmonic metasurface shown in Supplementary Fig. 5 is discussed in Supplementary Note 3.C. Finally, we give a proof of the equivalence between two alternative normalization for the plasmonic metasurface (Supplementary Note 3.D).

A. Definition and Notations

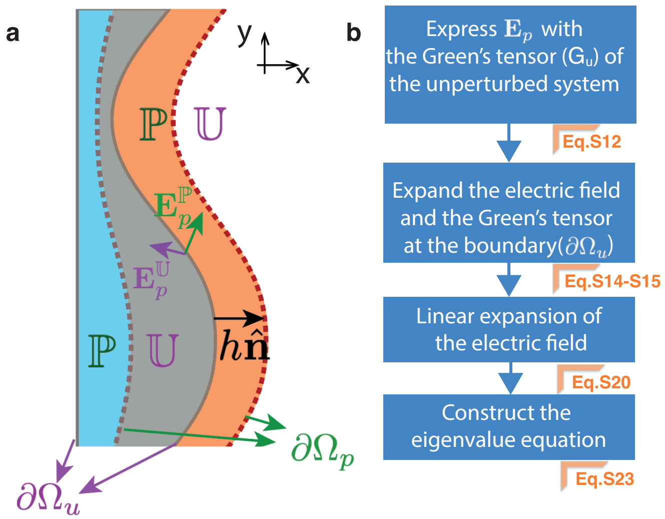

Supplementary Fig. 5. (a) Schematic illustration of the perturbation theory framework. A metasurface is deformed with the boundary changing from $\partial\Omega_u$ to $\partial\Omega_p$. The entire space is classified into two parts: $\mathbb{U}$ denotes the domain where the material remains the same after the deformation; $\mathbb{P}$ denotes the domain where the material is changed. (b) Workflow of the construction of the Hamiltonian.

- The superscript of a symbol represents the domain, which tells whether the field is in the unperturbed domain ($\mathbb{U}$) or perturbed domain ($\mathbb{P}$). If the notation of the field is in the absence of the superscript, it denotes the field in the entire domain.
- The subscript u and p of a symbol represents whether the electric field refers to the unperturbed system or perturbed system.
- $\mathbf{r}$ refers to the coordinate in the unperturbed system.
- $\mathbf{E}_{p,nk}^{\mathbb{U}}$ and $\mathbf{E}_{p,nk}^{\mathbb{P}}$ denote the electric field at unperturbed domain and perturbed domain in the perturbed system, respectively. The absence of the superscript indicates the electric fields are defined in the whole domain, such as $\mathbf{E}_{p,nk}$ denoting the electric field in perturbed system for both the perturbed domain and the unperturbed domain. Here, n denotes the band index and k is the Bloch wave vector.
- $h\hat{\mathbf{n}}$ is the perpendicular shift from $\partial\Omega_u$ to $\partial\Omega_p$. $\hat{\mathbf{n}}$ is the normal vector of the unperturbed surface.
- $\epsilon_p(\boldsymbol{r})$ and $\epsilon_u(\boldsymbol{r})$ are the permittivity distributions of the perturbed system and the unperturbed system.
- The permittivity of the metasurface and the background medium is ϵ_m and ϵ_b $(= \epsilon_m - \Delta\epsilon)$, respectively.

B. Formulation of the Hamiltonian

The workflow of this method is shown in Supplementary Fig. 5(b). The construction of the Hamiltonian starts from the Lippman-Schwinger intergral equation, which expresses the electric fields of the perturbed system with the Green's tensor of the unperturbed system

$$\mathbf{E}_{p,nk}(\boldsymbol{r};\omega) = \omega^2 \iiint \mathbf{G}_u(\boldsymbol{r},\boldsymbol{r'};\omega)\left(\epsilon_p(\boldsymbol{r'};\omega) - \epsilon_u(\boldsymbol{r'};\omega)\right)\mathbf{E}_{p,nk}(\boldsymbol{r'})\mathrm{d}^3\boldsymbol{r'}. \tag{S13}$$

The integral is carried out for the entire space, however, in the unperturbed domain, $\epsilon_p(\boldsymbol{r'};\omega) - \epsilon_u(\boldsymbol{r'};\omega) = 0$. Here, the Green's tensor of the original system satisfies

$$\left[\nabla \times \mu_0^{-1}\nabla \times -\omega^2\epsilon_u(\boldsymbol{r}';\omega)\right]\mathbf{G}_u(\boldsymbol{r},\boldsymbol{r'};\omega) = \mathbf{I}\delta(\boldsymbol{r}-\boldsymbol{r}'). \tag{S14}$$

The electric field in the perturbed domain is extrapolated as the Taylor series expansions employing $\mathbf{E}^{\mathbb{U}}_{p,nk}$ at $\partial\Omega_u$

$$\mathbf{E}^{\mathbb{P}}_{p,nk}(\boldsymbol{r}) = \sum_{j=0}^{\infty}(l^j/j!)\overrightarrow{\partial_{\hat{\mathbf{n}}}}^{j}\mathbf{E}^{\mathbb{U}}_{p,nk}(\boldsymbol{r}_{\partial\Omega_u}), \tag{S15}$$

where $\boldsymbol{r} = \boldsymbol{r}_{\partial\Omega_u} + l\hat{\mathbf{n}}$. Meanwhile, $\mathbf{G}_u$ can be expand as Taylor series at unperturbed boundary

$$\mathbf{G}_u(r,r';\omega) = \sum_{q=0}^{\infty}\overrightarrow{\partial_{\hat{\mathbf{n}}}}^{q}\mathbf{G}_u(r,r'_{\partial\Omega_u};\omega)\frac{l^q}{q!}. \tag{S16}$$

By substituting Eqs. S15 and S16 into Eq. S13, we arrive at a surface integration equation

$$\mathbf{E}_{p,nk}(\boldsymbol{r};\omega) = \omega^2 \iint_{\partial\Omega_u} G_u(\boldsymbol{r},\boldsymbol{r'};\omega)\mathbf{P}_{Geom}(\boldsymbol{r}'_{\partial\Omega_u};\omega)da, \tag{S17}$$

with

$$\mathbf{P}_{Geom}(\boldsymbol{r}_{\partial\Omega_u};\omega) = \sum_{j=0}^{\infty}\sum_{q=0}^{\infty}\overleftarrow{\partial_{\hat{\mathbf{n}}}}^{q}c_{jq}(\boldsymbol{r}_{\partial\Omega_u})\Delta\epsilon\overrightarrow{\partial_{\hat{\mathbf{n}}}}^{j}\mathbf{E}^{\mathbb{U}}_{p,nk}(\boldsymbol{r}_{\partial\Omega_u}), \tag{S18}$$

and

$$c_{j,q} = \frac{h^{q+j+1}}{q!j!}\left[\frac{1}{j+q+1} + \kappa_m\frac{2h}{j+q+2} + \kappa_g\frac{h^2}{j+q+3}\right], \tag{S19}$$

where κ_m and κ_g are the mean curvature and the Gaussian curvature, which are defined by $\kappa_m = (\kappa_1 + \kappa_2)/2$ and $\kappa_g = \kappa_1\kappa_2$. Here, κ_1 and κ_2 are the two principle curvatures of the metasurface. The notation $F\overleftarrow{\partial_n}^{q}$ denotes $(\hat{\mathbf{n}}\cdot\nabla)^q F$.

Since the completeness of the eigenstates can be constructed by introducing the left eigenstates as detailed in Supplementary Note 3.D, the Green's tensor can be expanded in terms of the normalized eigenmodes and the corresponding adjoint eigenstates at eigenfrequency $\omega_{u,nk}$, which is given by

$$G_u(r,r';\omega) = -\sum_{n}\frac{\mathbf{E}_{u,nk}(\boldsymbol{r})\otimes\mathbf{E}^{\mathbb{P}*}_{u,nk}(\boldsymbol{r'})}{\omega(\omega-\omega_{u,nk})}. \tag{S20}$$

Note that the form of Green's function is similar with the open system case [4], except that the complex

conjugated of the field, which is due to the form of the left eigenstate in our metasurface system.

In the next step, we express the electric field in perturbed system with expansions of eigenmodes in the original system

$$\mathbf{E}_{p,mk} = \sum_n \alpha_n \mathbf{E}_{u,nk}. \tag{S21}$$

We now plug Eqs. S18 and S20 into the right-hand side of Eq.S17, and compare it with Eq. S21, we have

$$-\frac{\omega}{\omega - \omega_{\mathrm{u},nk}} \iint_{\partial\Omega_u} \mathbf{E}^{\mathbb{P}*}_{\mathrm{u},nk} \mathbf{P}_{\mathrm{Geom}} \left(\mathbf{r}_{\partial\Omega_u}; \omega\right) d^2\mathbf{r}_{\partial\Omega_u} = \alpha_n, \tag{S22}$$

where

$$\mathbf{P}_{\mathrm{Geom}} \left(\mathbf{r}_{\partial\Omega_u}; \omega\right) = \sum_n \sum_{j=0}^{\infty} \sum_{q=0}^{\infty} \alpha_n \overleftarrow{\partial}^q_{\hat{\mathbf{n}}} c_{jq} \left(\mathbf{r}_{\partial\Omega_u}\right) \vec{\partial}^j_{\hat{\mathbf{n}}} \Delta\epsilon \mathbf{E}^{\mathbb{U}}_{\mathrm{u},nk}. \tag{S23}$$

By defining $|\alpha\rangle = [\alpha_1; \alpha_2; ......; \alpha_n]$, we arrive at an eigenvalue equation

$$\left[\mathbf{I} + \mathcal{H}_p\right]^{-1} \mathcal{H}_0 |\alpha\rangle = \omega |\alpha\rangle, \tag{S24}$$

with $\mathcal{H}_{0;nm} = \omega_{u,nk}\delta_{nm}$, and

$$\mathcal{H}_{p;nm} = \sum_{j=0}^{\infty} \sum_{q=0}^{\infty} \langle \mathbf{E}^{\mathbb{P}}_{u,nk} | \overleftarrow{\partial_n}^q \Delta\epsilon c_{j,q} \overrightarrow{\partial_n}^j | \mathbf{E}^{\mathbb{U}}_{u,mk} \rangle_{\partial\Omega_u}. \tag{S25}$$

It should be mentioned again that the form of the Hamiltonian is slightly different from the reference [4], due to the complex conjugated state should be involved, resulting from the left eigenstate of our system.

C. Understanding the band inversion from the Hamiltonian approach

In this subsection, we apply the above Hamiltonian approach to analyze the band topology of our metasurface, and explain why sign change of a_0 of the conformal mapping can lead to band inversion. Here, we treat $a_0 = 0$

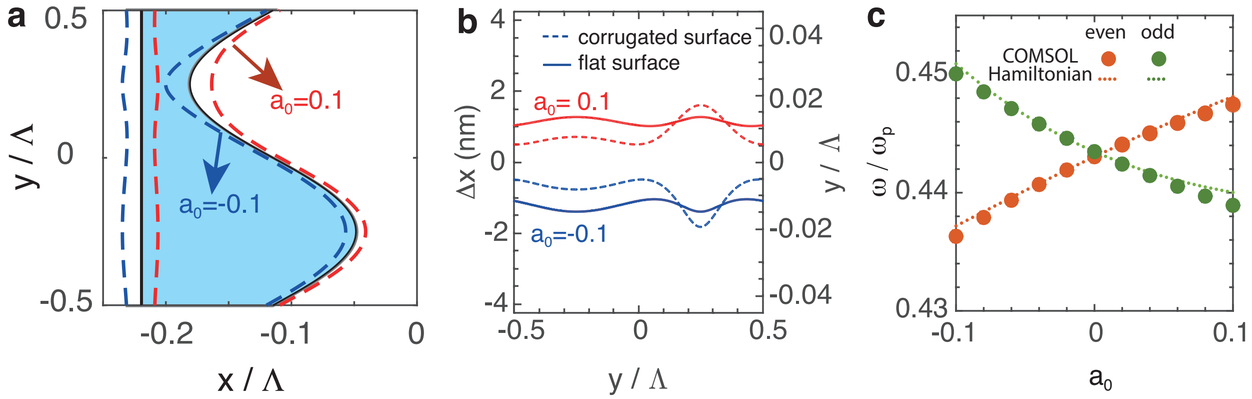

Supplementary Fig. 6. (a) Geometry of the metasurfaces with $a_0 = 0$(solid lines), $a_0 = -0.1$(dashed blue lines) and $a_0 = 0.1$(dahsed red lines), respectively. (b) Shape deformation of two surfaces with $a_0 = \pm 0.1$. (c) Eigenfrequecies of the metasurfaces with a_0 ranging from -0.1 to 0.1 calculated from COMSOL and the Hamiltonian approach, implying good agreement between two methods.

case as the unperturbed system, and the plasmonic system with a non-zero a_0 refers to the perturbed system. In our analysis, we deploy the eigenstates of the lower band and the upper band in consideration to constitute a two-band Hamiltonian, and only keep the first term ($j = 0$ and $q = 0$) in the Taylor expansion in Eqs. S15 and S16. The 2×2 Hamiltonian can then be written as

$$\mathcal{H} = [\mathbf{I} + \mathcal{H}_p]^{-1} \cdot \mathcal{H}_0, \tag{S26}$$

with

$$\mathcal{H}_{p,11} = \langle \mathbf{E}^{\mathbb{P}}_{u,1k} | h\Delta\epsilon | \mathbf{E}^{\mathbb{U}}_{u,1k} \rangle_{\partial\Omega_u}, \tag{S27}$$

$$\mathcal{H}_{p,22} = \langle \mathbf{E}^{\mathbb{P}}_{u,2k} | h\Delta\epsilon | \mathbf{E}^{\mathbb{U}}_{u,2k} \rangle_{\partial\Omega_u}, \tag{S28}$$

$$\mathcal{H}_{p,12} = \langle \mathbf{E}^{\mathbb{P}}_{u,1k} | h\Delta\epsilon | \mathbf{E}^{\mathbb{U}}_{u,2k} \rangle_{\partial\Omega_u}, \tag{S29}$$

and

$$\mathcal{H}_{p,21} = \langle \mathbf{E}^{\mathbb{P}}_{u,2k} | h\Delta\epsilon | \mathbf{E}^{\mathbb{U}}_{u,1k} \rangle_{\partial\Omega_u}, \tag{S30}$$

where $\langle \mathbf{E}^{\mathbb{P}}_{u,nk} | h\Delta\epsilon | \mathbf{E}^{\mathbb{U}}_{u,mk} \rangle_{\partial\Omega_u} = \int_{\partial\Omega_u} \mathbf{E}^{\mathbb{P}*}_{u,nk} \cdot h\Delta\epsilon \cdot \mathbf{E}^{\mathbb{U}}_{u,mk} d^2\mathbf{r}$. The electric field of the lower band and the upper band in the perturbed domain with the Bloch wave vector k_y are denoted by $\mathbf{E}^{\mathbb{P}}_{u,1k}$ and $\mathbf{E}^{\mathbb{P}}_{u,2k}$, respectively. If the field is reside in the unperturbed domain, the superscript changes to $\mathbb{U}$. Here, h is a function of $\mathbf{r}$. The expression of $\mathcal{H}_0$ is

$$\mathcal{H}_0 = \begin{pmatrix} \omega_{u,1k} & 0 \\ 0 & \omega_{u,2k} \end{pmatrix}, \tag{S31}$$

with $\omega_{u,1k}$ and $\omega_{u,2k}$ being the eigenfrequencies for the lower band and the upper band of the unperturbed system.

Assume $\mathcal{H}_{p,11}, \mathcal{H}_{p,22}, \mathcal{H}_{p,12}, \mathcal{H}_{p,21} \ll 1$, and Eq. S26 can be written as

$$\mathcal{H} = \begin{pmatrix} \omega_{u,1k}(1 + \mathcal{H}_{p,22}) & -\omega_{u,2k}\mathcal{H}_{p,12} \\ -\omega_{u,1k}\mathcal{H}_{p,21} & \omega_{u,2k}(1 + \mathcal{H}_{p,11}) \end{pmatrix}. \tag{S32}$$

We then apply the following rotation

$$U = \begin{pmatrix} 1/\sqrt{2} & -1/\sqrt{2} \\ +1/\sqrt{2} & 1/\sqrt{2} \end{pmatrix}, \tag{S33}$$

to Eq. S32 as $H = U^{-1}\mathcal{H}U$, and the Hamiltonian becomes

$$H = (\frac{\omega_{u,1k} + \omega_{u,2k}}{2} + \Delta_{A+})I + (\frac{\omega_{u,2k} - \omega_{u,1k}}{2} + \Delta_{A-})\sigma_x + \mathrm{i}\Delta_{B-}\sigma_y - \Delta_{B+}\sigma_z, \tag{S34}$$

with $\sigma_x = \begin{pmatrix} 0 & 1 \\ 1 & 0 \end{pmatrix}$, $\sigma_y = \begin{pmatrix} 0 & -\mathrm{i} \\ \mathrm{i} & 0 \end{pmatrix}$ and $\sigma_z = \begin{pmatrix} 1 & 0 \\ 0 & -1 \end{pmatrix}$, and

$$\Delta_{A\pm} = \frac{\omega_{u,1k}\mathcal{H}_{p,11} \pm \omega_{u,2k}\mathcal{H}_{p,22}}{2}, \tag{S35}$$

and

$$\Delta_{B\pm} = \frac{\omega_{u,1k}\mathcal{H}_{p,21} \pm \omega_{u,2k}\mathcal{H}_{p,12}}{2}. \tag{S36}$$

At the Γ point, the two states are degenerated at ω_0, and they possess the opposite symmetry. Therefore, if a_0 is a real number, the Hamiltonian Eq. S34 can be simplified to

$$H = \omega_0 \left[(1 + \Delta_+) \, \mathrm{I} + \Delta_- \sigma_x \right], \tag{S37}$$

where

$$\Delta_{\pm} = \frac{\mathcal{H}_{p,11} \pm \mathcal{H}_{p,22}}{2}. \tag{S38}$$

The difference between $\mathcal{H}_{p,11}$ and $\mathcal{H}_{p,22}$ acts as the interaction, while the average of them plays the role of self-energy. The sign change of Δ_- can drive a topological transition in our system.

We first explain why sign change of a_0 can lead to band inversion. The geometries of the metasurfaces with different a_0 imposed are shown in Supplementary Fig. 6a, and the corresponding shape deformations of the two surfaces compared with $a_0 = 0$ are shown in Supplementary Fig. 6b. The geometric deformations for both the corrugated and the flat surface with a_0 changing from -0.1 to 0.1 are depicted in Supplementary Fig. 7, implying the sign change of a_0 will cause the deformation switching to the opposite direction. If we assume that the deformations with opposite a_0 share the same perturbed depth but possess an opposite sign, which means that only the sign of h is changed, this will lead to the sign change of the integrals in Eqs. S27 and S28. Therefore, the sign change of a_0 will result in the sign change of Δ_-, and thus gives rise to the band inversion. Shown in Supplementary Fig. 6c are the eigenfrequencies at the Γ point as a function of a_0 obtained from the Hamiltonian approach and the eigenvalue solver in COMSOL, which are in good agreement.

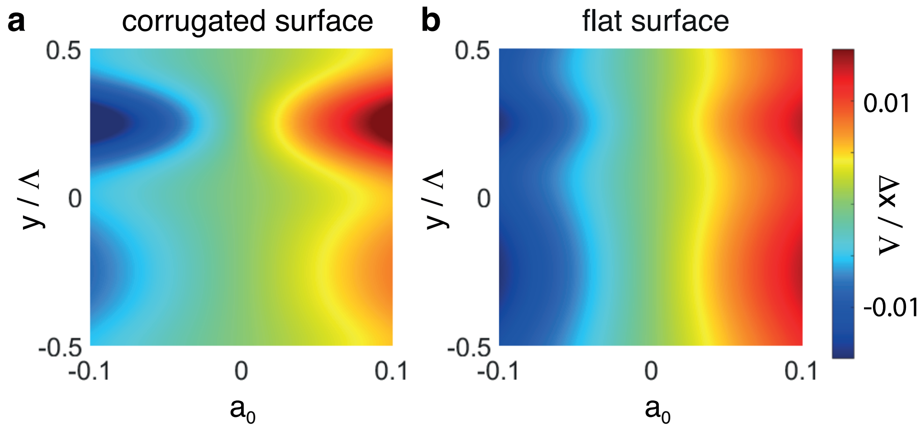

Supplementary Fig. 7. Deformation in the unit of Λ on the (a) corrugated surface and on the (b) flat surface. Here, Λ is 94.2 nm in our calculation.

As shown in Supplementary Fig. 6c, the band gap centers are almost at the gap closing frequency with different a_0 imposed. In the next step, we explain the fixing of the band gap center with the Hamiltonian approach. In Supplementary Fig. 8a, Δ_+ for the two surfaces are separately discussed, *i.e.* the blue solid line is the contribution from the corrugated surface, and the orange solid line is the contribution from the flat surface. As shown in Supplementary Fig. 8a, the values of Δ_+ of the two surfaces cancel with each other. As illustrated in Eq. S37, Δ_+ represents the self-energy, and thus the perturbation will retain the gap center in our case. As for the values of Δ_- of the two surfaces, the corrugated surface contribution dominates, and the sum of the Δ_- for the two surfaces is no longer zero, resulting in the band inversion.

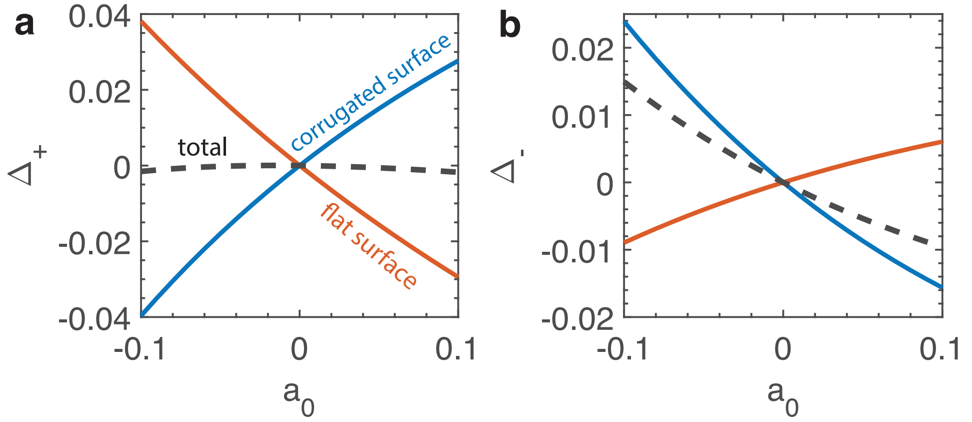

Supplementary Fig. 8. (a) Δ_+ of the first corrugated surface (solid blue line), the second flat surface (solid orange line), and the sum of the Δ_+ of the two surfaces (dashed gray line). (b) Δ_- of the first corrugated surface (solid blue line), the second flat surface (solid orange line), and the sum of the Δ_- of the two surfaces (dashed gray line).

D. Orthogonality of the eigenstates

This subsection provides the derivation of the orthogonality relation of the eigenstates for a dispersive system. For source-free Maxwell equations with auxiliary fields, the eigenequation reads as

$$\hat{\mathbf{H}}(\mathbf{r})\Psi(\omega,\mathbf{r}) = \omega\Psi(\omega,\mathbf{r}), \tag{S39}$$

with

$$\hat{\mathbf{H}}(\mathbf{r}) = \begin{bmatrix} 0 & -i\mu_0^{-1}\nabla\times & 0 & 0 \\ i\varepsilon_\infty^{-1}\nabla\times & 0 & 0 & -i\varepsilon_\infty^{-1} \\ 0 & 0 & 0 & i \\ 0 & i\omega_p^2(\mathbf{r})\varepsilon_\infty & -i\omega_0^2(\mathbf{r}) & -i\gamma(\mathbf{r}) \end{bmatrix}, \tag{S40}$$

and

$$\Psi(\omega,\mathbf{r}) = [\mathbf{H}(\omega,\mathbf{r}), \mathbf{E}(\omega,\mathbf{r}), \mathbf{P}(\omega,\mathbf{r}), \mathbf{J}(\omega,\mathbf{r})]^{\mathrm{T}}. \tag{S41}$$

The completeness relation for eigenstates of a linear non-Hermitian problem can be constructed by introducing the eigenstates of the complex conjugated transposes of the initial eigen-operator [5]. Another interpretation is that we should use the left eigenstates to form the bi-orthogonal set. As we set out to prove, these two approaches are equivalent in this framework.

Define $\hat{\mathbf{D}} = \mathrm{diag}\left[-\mu_0, \varepsilon_\infty, \omega_0^2/\left(\varepsilon_\infty\omega_p^2\right), -1/\left(\varepsilon_\infty\omega_p^2\right)\right]$, and ignore the loss ($\gamma = 0$), then we have

$$\hat{\mathbf{H}}^\dagger\hat{\mathbf{D}} = \hat{\mathbf{D}}\hat{\mathbf{H}}^*. \tag{S42}$$

Multiplying $\hat{\mathbf{D}}^{-1}$ to each side, and taking the complex conjugate, we have

$$\hat{\mathbf{H}}^T = \hat{\mathbf{D}}\hat{\mathbf{H}}\hat{\mathbf{D}}^{-1}. \tag{S43}$$

For the non-Hermitian photonic crystals, the corresponding left eigenstates can be defined as

$$H^\dagger\Psi_k^L = \omega^*\Psi_k^L. \tag{S44}$$

Taking complex conjugate of Eq. S44 and multiplying D^{-1}, we obtain

$$\hat{\mathbf{D}}^{-1}(\hat{\mathbf{H}}^{\dagger}\Psi_k^L)^* = \hat{\mathbf{H}}\hat{\mathbf{D}}^{-1}\Psi_k^{L*} = \hat{\mathbf{D}}^{-1}\omega\Psi_k^{L*}, \tag{S45}$$

which means that $\hat{\mathbf{D}}^{-1}\Psi_k^{L*}$ is a right eigenstate of $\hat{\mathbf{H}}$.

We then can prove that Ψ_k^L is an Bloch state at k. Using translation operator $\mathbf{T}$ to operate on $\hat{\mathbf{D}}^{-1}\Psi_k^{L*}$, we arrive at

$$\mathbf{T}\hat{\mathbf{D}}^{-1}\Psi_k^{L*}(\mathbf{r}) = \hat{\mathbf{D}}^{-1}\Psi_k^{L*}(\mathbf{r}+\mathbf{a}) = \mathrm{e}^{-ika}\hat{\mathbf{D}}^{-1}\Psi_k^{L*}(\mathbf{r}). \tag{S46}$$

Thus, $\hat{\mathbf{D}}^{-1}\Psi_k^{L*}(\mathbf{r})$ is the right Bloch state at $-\mathbf{k}$, which further indicates

$$\Psi_k^L = (\hat{\mathbf{D}}\Psi_{-k}^R(\mathbf{r}))^*. \tag{S47}$$

Equation S47 shows that the left eigenstate of a dispersive photonic crystal can be obtained by using the right eigenstate at its reciprocal k point. It is worth pointing out that Eq. S47 recovers $\mathbf{E}_k^L = (\epsilon\mathbf{E}_{-k}^R)^*$ for a dispersionless non-Hermitian photonic crystal [6].

The bi-orthogonal relation of a non-Hermitian photonic crystal can be expressed as

$$\int \Psi_{-kn}^R\hat{\mathbf{D}}\Psi_{km}^R \mathrm{d}^3r = \delta_{nm}. \tag{S48}$$

Note that our system also possesses the time reversal symmetry, and thus the Bloch states of the electric field satisfy

$$\mathbf{u}_{-kn}(\mathbf{r}) = \mathbf{u}_{kn}(\mathbf{r})^*. \tag{S49}$$

The electric field at $-k$ can be written as

$$\mathbf{E}_{-kn} = \mathbf{u}_{-kn}e^{-ikr} = \mathbf{u}_{kn}^*e^{-ikr}. \tag{S50}$$

Therefore, we have

$$\mathbf{E}_{kn} = \mathbf{E}_{-kn}^*. \tag{S51}$$

The magnetic field can be obtained via

$$\nabla\times\mathbf{E}(\mathbf{r},t) = i\omega\mu_0\mathbf{H}(\mathbf{r},t), \tag{S52}$$

which indicates

$$\mathbf{H}_{-kn} = -\mathbf{H}_{kn}^*. \tag{S53}$$

Similar analysis can be applied to $\mathbf{P}$ and $\mathbf{J}$, and finally we have

$$\Psi_{-kn} = [-\mathbf{H}_{kn}^*, \mathbf{E}_{kn}^*, \mathbf{P}_{kn}^*, -\mathbf{J}_{kn}^*]. \tag{S54}$$

By substituting Eq. S54 to Eq. S48, we arrive at

$$\int\left(\epsilon_\infty\mathbf{E}_{km}\mathbf{E}_{kn}^* + \mu_0\mathbf{H}_{km}\mathbf{H}_{kn}^* + \frac{\omega_0^2}{\varepsilon_\infty\omega_p^2}\mathbf{P}_{km}\mathbf{P}_{kn}^* + \frac{1}{\varepsilon_\infty\omega_p^2}\mathbf{J}_{km}\mathbf{J}_{kn}^*\right)d^2\mathbf{r} = \delta_{nm}. \tag{S55}$$

This recovers the orthogonal relation dealing with the lossless dispersive photonic crystal system [7]. In summary, for a reciprocal system, we can always use the right eigenstate at $-k$ to form the bi-orthogonal set at $+k$, namely Eq. S48. Furthermore, if the system also possesses the time reversal symmetry, Eq. S48 recovers the known results for the Hermitian dispersive crystals, namely Eq. S55. Since we use the Drude/Lorentz model to describe our system, which are both reciprocal and time-reversal symmetric, Eqs. S48 and S55 are equivalent.

Supplementary Note 4. CORRELATION OF THE EIGENSTATES BETWEEN TWO SPACES

A. The band structures of the plasmonic system in the virtual space

The plasmonic system in the virtual space consists of a slab and an eccentric cylinder. The slab is positioned at $u = u_0\Lambda/[2\pi]$ with the thickness $d\Lambda/[2\pi]$. Here, the scale factor $\Lambda/[2\pi]$ is applied to the geometry in the virtual space, therefore, the lattice constant is the same for the plasmonic systems in two spaces. The parameters are $u_0 = 1$ and $d = 0.5$. The geometry of the eccentric cylinder is determined by the inverse transform illustrated in Eq. S4. The Drude model is used with $\omega_p = 2$ eV, and the system is considered to be lossless. The band structures for the plasmonic system with $a_0 = \pm 0.1$ are shown in Supplementary Fig. 9, illustrating the increasing of the dimension in the virtual space and two sets of double fold degenerated states at the Γ point.

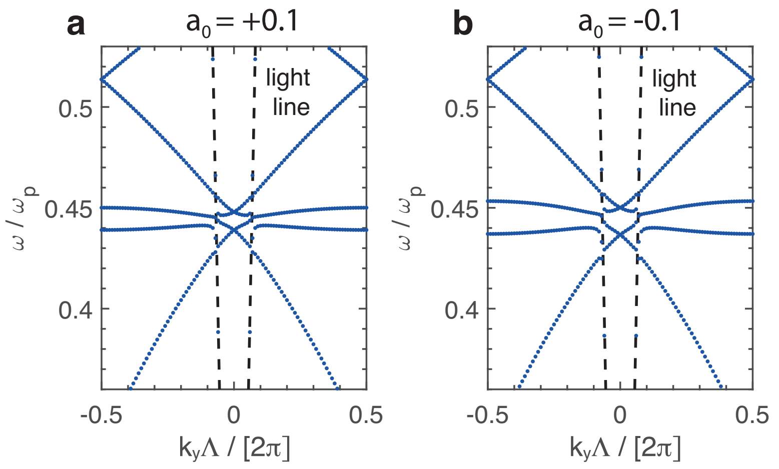

Supplementary Fig. 9. Band strucutres for the plamsonic systems with $a_0 = +0.1$(a) and $a_0 = -0.1$(b) in the virtual space.

B. Projection of the eigenstates

In this subsection, we investigate the eigenstates at the Γ point in the virtual space, and illustrate the correlations of the eigenstates between the two spaces. Supplementary Figs. 10a-h show the magnetic field distributions of the eigenmodes at the Γ point in the virtual space. It can be inferred that for each two degenerated states, there are an even mode and an odd mode. If the metasurface in the real space has mirror symmetry, TO will directly pick up one of the two states from the virtual space due to the conservation of the potential. For $a_0 = +0.1$ case, the odd mode is projected to the real space for the lower band, meanwhile, the even mode is selected for the upper band, constituting a topologically trivial system in the real space. However, as for the $a_0 = -0.1$ case, the even mode is projected to the real space for the lower band, and the odd mode is at the upper band of the metasurface in the real space, resulting in a topologically non-trivial system. Therefore, our projection method depicts how a topologically non-trivial system can be constructed from a higher dimensional

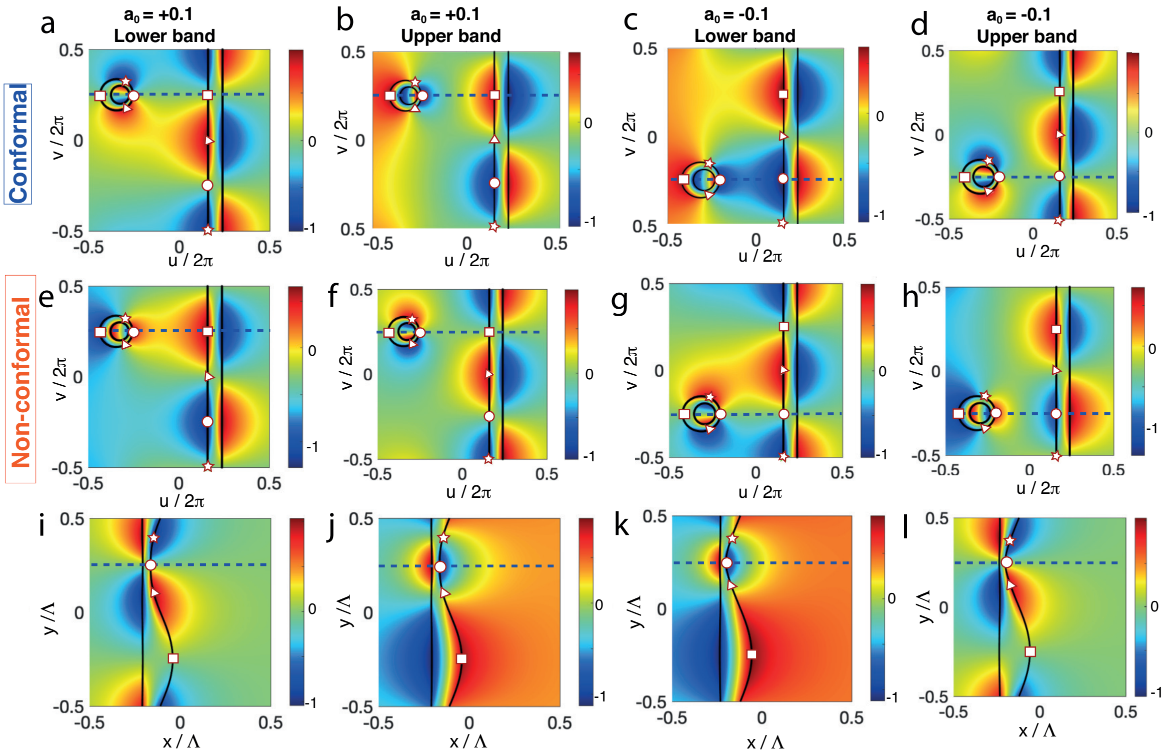

Supplementary Fig. 10. (a)-(h) show the Im(H_z) distributions for the eigenstates in the virtual space. (i)-(l) shown the Im(H_z) distributions for the eigenstates in the real space. The parameters of the metasurfaces are consistent with the metasurface demonstrated in Figs. 3a-d in the main text. The dashed lines denote the symmetry plane, and the circle, triangle, star and square markers share the same notation with Figure 1 in the main text.

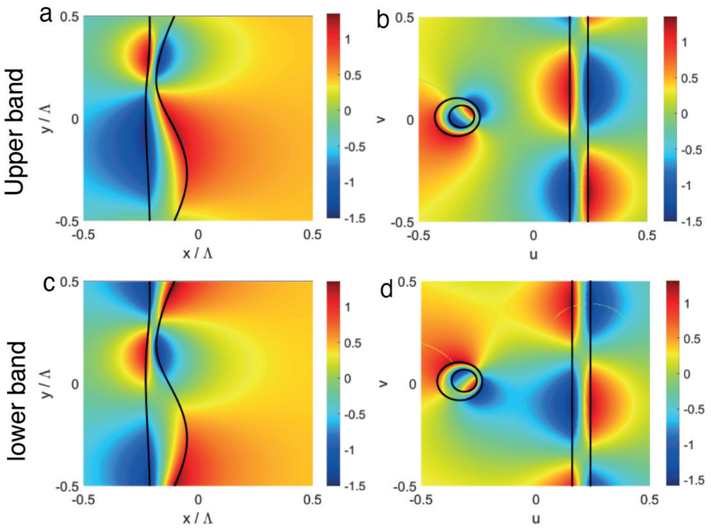

Supplementary Fig. 11. (a) and (c) show the Im(H_z) distributions for the eigenstates in the real space with $a_0 = 0.1\exp(-i\pi/2)$, and the transformed field distributions in the virtual space are shown in (b) and (d), respectively.

system.

While a_0 is a complex value, the mirror symmetry of the metasurface is broken in the real space. The projection of the eigenstates in the virtual space will involve a linear combination of the two degenerated states. Shown in Supplementary Figs. 11a and c are the magnetic field distributions of the states for the lower and the upper

band in the real space with $a_0 = 0.1\exp(-i\pi/2)$, and we then transform the magnetic field to the virtual space as shown in Supplementary Figs.11b and d, demonstrating the projection of the states when a_0 is a complex value.

Supplementary Note 5. CALCULATION OF THE WANNIER FUNCTIONS

Since the eigenvectors obtained by numerical diagonalization may contain random phases, a smooth gauge procedure should be carried out to guarantee the parallel transport of the eigenvectors. In this section, we discuss the methodology which can be found in the Ref. [8].

In the first step, we uniformly discretize the k points in the first Brillouin zone (BZ), and obtain the corresponding states $|u_{k_n}\rangle$, here $|u_{k_n}\rangle = (\mathbf{H}, \mathbf{E}, \mathbf{P}, \mathbf{J})^T$. At the initial point, we set $|u'_{k_1}\rangle = |\tilde{u}_{k_1}\rangle$, then apply the phase rotation to the state at the next k point,

$$|u'_{k_{j+1}}\rangle = e^{i\beta_{j+1}}|\tilde{u}_{k_{j+1}}\rangle, \tag{S56}$$

where $\beta_{j+1} = \mathrm{Im}\ln\langle\tilde{u}_{k\,j+1}|\tilde{u}_{k_j}\rangle$. After carrying out the rotation to all the states, the state at k_1 differs from k_{N+1} by a phase factor $e^{\mathrm{i}\phi}$, which is the Zak phase of the metasurface. To restore the periodicity in k_y, we add an extra phase to each state,

$$|u_{k_j}\rangle = e^{-i\phi k_j/2\pi}|u'_{k_j}\rangle. \tag{S57}$$

After carrying the smooth gauge for all the states in the 1st BZ, the Wannier function can be readily obtained, which reads

$$|\mathbf{R}_n\rangle = \frac{V}{(2\pi)^3}\int_{\mathrm{BZ}} d\mathbf{k}e^{-i\mathbf{k}\cdot\mathbf{R}}\,|u_{n\mathbf{k}}\rangle\,. \tag{S58}$$

Here, $|\mathbf{R}_n\rangle$ refers to the Wannier function associated with band n.

Supplementary Note 6. DISCUSSION ON THE HIGHER ORDER BANDS

In this section, we show that our framework can be applied to the bands with higher band index. The band structures for the metasurfaces with $a_0 = +0.1$ and $a_0 = -0.1$ are shown in Supplementary Fig. 12. From the marked Zak phases, it can be inferred that the interface state can exist in the first band gap of the two metasurfaces, and cannot exist in the second band gap at the BZ center marked in magenta.

As shown in Supplementary Figs. 13a, b, e, and f, the symmetries of the eigenstates at the Γ point are the same for $a_0 = \pm 0.1$ with the identical band index, implying the band will not invert with sign change of a_0 for the third and the forth bands. Not only can the existence of the interface state be implied by the symmetries of the eigenmodes at the Γ point of the corresponding bands, in the next step, we give an explanation from the virtual space. The TO-enabled states in the virtual space are also shown in Supplementary Fig. 13. The even mode is positioned at the lower band due to the attraction between the cylinder and the slab for both $a_0 = \pm 0.1$ cases, as shown in Supplementary Figs. 13d and h. And the odd mode is at the higher frequency due to the repulsive force, as shown in Supplementary Figs. 13c and g.

The shift of the Wannier function center (WFC) should be in the unit of the lattice constant Λ. For the third and the forth band, the WFC shifts 2Λ following the opposite directions, as shown in Supplementary Fig. 14.

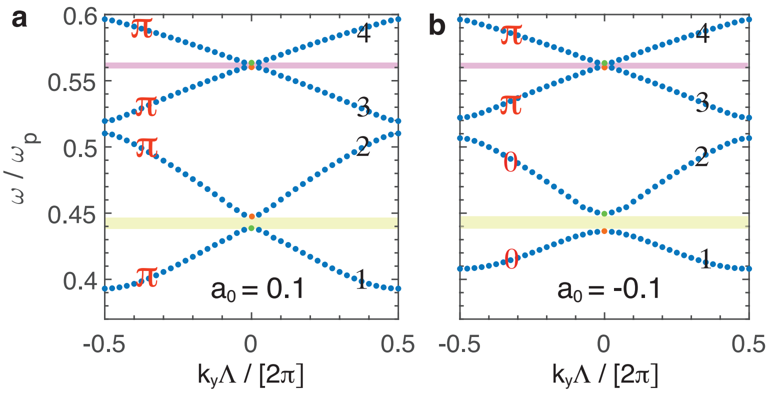

Supplementary Fig. 12. The band structures for the metasurfaces with (a) $a_0 = -0.1$ and (b) $a_0 = +0.1$. The yellow strip denotes the first band gap, and the magenta strip denote the second band gap at the BZ center. The Zak phase of each individual band is labeled in red. The band index is labeled in black, where the lowest frequency band is defined as band-0.

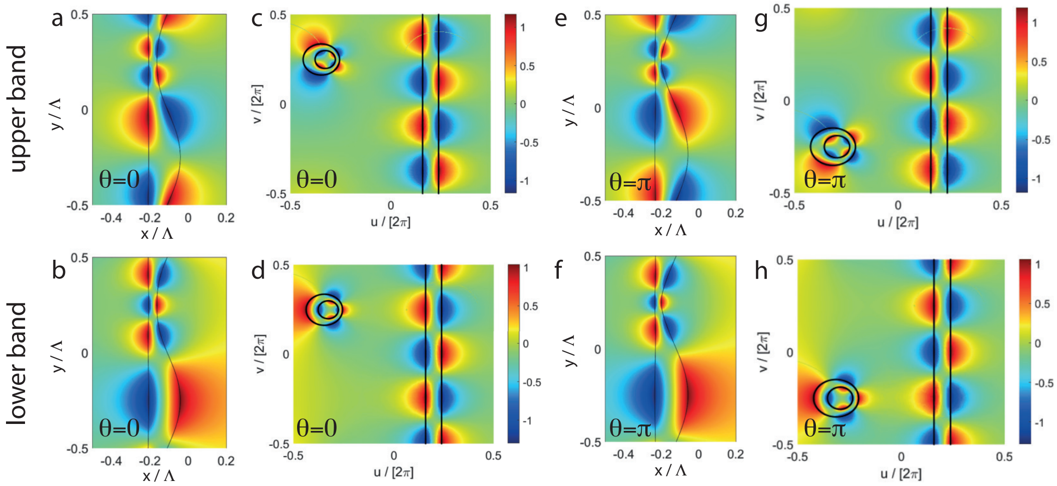

Supplementary Fig. 13. The calculated $\mathrm{Im}(H_z)$ distributions for the two eigenstates at the Γ point for the third and the forth band in the real space (a, b, e, and f) and in the virtual space (c, d, g, and h).

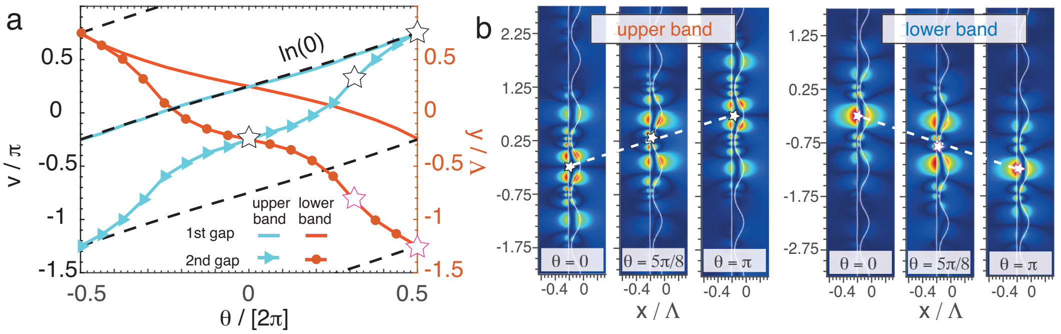

Supplementary Fig. 14. (a) The cyan and orange solid lines depict the y position of the Wannier function centers in the real space for the lower and upper band, respectively. The v coordinates of the ln(0) singularities as a function of θ are shown by the dashed lines. (b) The Wannier functions $|H_z|$ for the lower and the upper bands with $\theta = 0, 5\pi/8$ and π.

[1] P. Huidobro, Y. Chang, M. Kraft, and J. Pendry, Hidden symmetries in plasmonic gratings, Physical Review B **95**, 155401 (2017).

[2] J. Pendry, P. A. Huidobro, and K. Ding, Computing one-dimensional metasurfaces, Physical Review B **99**, 085408 (2019).

[3] J. B. Pendry, P. A. Huidobro, and K. Ding, Computing one-dimensional metasurfaces, Physical review. B, Condensed Matter And Materals Physics **99**, 085408.1 (2019).

[4] W. Yan, P. Lalanne, and M. Qiu, Shape deformation of nanoresonator: a quasinormal-mode perturbation theory, Physical Review Letters **125**, 013901 (2020).

[5] W. Yan, R. Faggiani, and P. Lalanne, Rigorous modal analysis of plasmonic nanoresonators, Physical Review B **97**, 205422 (2018).

[6] X. Cui, K. Ding, J.-W. Dong, and C. T. Chan, Exceptional points and their coalescence of pt-symmetric interface states in photonic crystals, Physical Review B **100**, 115412 (2019).

[7] A. Raman and S. Fan, Photonic band structure of dispersive metamaterials formulated as a hermitian eigenvalue problem, Physical Review Letters **104**, 087401 (2010).

[8] A. A. Soluyanov and D. Vanderbilt, Smooth gauge for topological insulators, Physical Review B Condensed Matter **85**, 777 (2012).
