# Supplementary material for: Revealing topology with transformation optics
Source: Nat Commun. 2021 Nov 25;12:6887. doi: 10.1038/s41467-021-27008-x (PMC8617177; doi:10.1038/s41467-021-27008-x)
Supplement: Supplementary file 1 — Author Checklist [file 41467_2021_27008_MOESM1_ESM.docx]

|  |  |
| --- | --- |
| **Author Checklist** | NCOMMS-NCOMMS-21-33978-T |
| **0000000000000000000000000000000000000000000000000000000000000** | **0000000000000000000000000000000000000** |
| **Please check the items below carefully and add a response in each row of the table to indicate the changes that you have made. Please also check through any additional marked-up edits we have may have provided within the manuscript file.** | |
|  |  |
|  |  |
|  |  |
| Abstract and editor's summary |  |
| Our guidance: | Your response: |
| Your paper will be accompanied by the following editor's summary. Please let us know if there are any inaccuracies: 'Visualizing and studying topological features is becoming crucial to develop new generation topological systems. Here the authors provide the theoretical and numerical demonstration of the potential of transformation optics connecting the virtual space to the physical space of lower dimension via conformal mapping.' | We thank the editor for the summary and are happy with that. |
| Please shorten the abstract to 150 words or fewer. | The abstract has been shortened. |
|  |  |
| Author information |  |
| Our guidance: | Your response: |
| Please review your complete author list to verify that it is complete and accurate. We ask that you consult with your coauthors to ensure that all names, affiliations, and titles are represented correctly. Note that if any authors are added or removed after this point then all authors will be requested to provide approval documentation that could potentially delay the production of your paper. | One of the co-authors of our paper, Emanuele Galiffi, has moved to City University of New York. He is now affiliated to both Imperial College London and City University of New York. Therefore, we have updated his affiliation to the revised manuscript. Other details of the author list are correct. |
|  |  |
| Article structure |  |
| Our guidance: | Your response: |
| We can accommodate up to 10 display items (Figures or Tables) in the main article. Each Figure and Table must fit easily within an A4 page (210 x 297 mm). Please ensure that the number and size of your Figures and Tables fulfil these requirements to avoid any delay in the acceptance of your article. | The display items have been checked. |
| **To comply with this format and optimise the presentation of data in your Article, we suggest the following changes to the display items in your paper:** |  |
| *Please revise the figures reference in the main text. Sometimes is refereed to [panel x] instead of the full figure number, i.e. Fig. 3x,* | *We have changed the figures reference to the full figure number, i.e., Fig. 3x.* |
|  |  |
| *Title Author list Affiliations Abstract Introduction Results Discussion (optional) Results and Discussion (optional) Methods (including Data Availability, Code Availability and Statistics subsections where relevant) References Acknowledgements Author Contributions Statement Competing Interests Statement Tables Figure Legends/Captions (for main text figures)* | *The manuscript has been organized as suggested.* |
| We do not edit Supplementary Information files; they will be uploaded with the published article as they are submitted with the final version of your manuscript. Any tracked changes should be removed from the file and the file should be provided as a PDF file. Supplementary Figures do not need to be provided separately. |  |
|  |  |
|  |  |
| Main text |  |
| Our guidance: | Your response: |
| Please rearrange the Introduction so that all discussion of previous work appears first. The final paragraph should contain only a concise summary of the current work, in the present tense, and begin with a phrase like “In this work” or “Here, we show”. | The introduction has been rearranged, and the final paragraph has been revised with the summary of the current work. |
| Please divide the Results section into subsections, each with a title of 60 characters or fewer including spaces. | The Results section has been divided into subsections. |
| Please provide a Discussion section in the main manuscript file. | A discussion section has been provided. |
| Please do not use italics, bold font, underlining or speech marks unless required for technical terms (in both the main text and the display items). | We have checked those issues. |
| Please make sure that mathematical terms throughout your manuscript and Supplementary Information (including in figures, figure axes, and legends) conform strictly to the following guidelines. Equations must be supplied in editable format, and not as images. Scalar variables (e.g. x, V, χ) must be typeset in italic, whereas multi-letter variables and functions (e.g. log) must be formatted in roman. Vectors (such as the wavevector k or the magnetic field vector B) must be typeset in bold without italics. | We have checked the mathematical terms throughout the manuscript and Supplementary Information. |
|  |  |
| Figures and Tables |  |
| Our guidance: | Your response: |
| Please see the guidelines linked below for detailed instructions about how your figures should be prepared. Following these instructions will reduce the chances of delays should we need to request replacement artwork from you at a later stage. | We have checked the guidelines, and the figures in pdf format are provided as separated files. |
| <https://www.nature.com/documents/NRJs-guide-to-preparing-final-artwork.pdf> |  |
| All colour scales must be defined and intensity levels must be provided in either the figure or its associated legend. Please provide labels to these legends, e.g. at colour bars, in the format “variable [unit]” or similar. | Figures 1-3 are in the correct format. We have added the color bars in fig.4, indicating the intensity levels. |
| Shadings or symbols in graphs must be defined in some fashion. We prefer that you use a key within the image; do not include colored symbols in the legend/caption. | We have checked the symbols in the graphs, which have been defined. |
| Any abbreviations, symbols or colours present in your figures must be defined in the associated legends. | We have checked abbreviations, symbols or colours in the figures have been defined. |
|  |  |
| Data and Code |  |
| Our guidance: | Your response: |
| Nature journals strongly support public availability of data and code. Please deposit the data and code used in your paper into a public data repository, or alternatively, present the data as Supplementary Information. If data can only be shared on request, please explain why in your Data Availability Statement, and also in the correspondence with your editor.   Please note that for some data types, deposition in a public repository is mandatory. Any restrictions on sharing of these data types must be clearly indicated in the statement and discussed with the editor. More information on our data deposition policies and available repositories can be found here: | The data in our work can be shared upon request. Since all the necessary parameters have been put down in the manuscript and the numerical packages are easy to access by the readers, we choose to share our raw data upon request. |
| <https://www.nature.com/nature-research/editorial-policies/reporting-standards#availability-of-data> |  |
| All published manuscripts reporting original research in Nature Portfolio journals must include a data availability statement, as a separate section before the References and under the heading 'Data Availability'.   The data availability statement must make the conditions of access to the “minimum dataset” that are necessary to interpret, verify and extend the research in the article, transparent to readers.   This minimum dataset may be provided through deposition in public community/discipline-specific repositories, custom proprietary repositories or general repositories like Figshare, Zenodo and Dryad. Providing large datasets in supplementary information is strongly discouraged and the preferred approach is to make data available in repositories. Scientific Data, a Nature Portfolio journal, maintains a list of approved and recommended data repositories to support researchers seeking suitable repositories for their data (https://www.nature.com/sdata/policies/repositories).  The Data Availability Statement should also reference any source data published alongside the paper.  If DOIs are provided, we also strongly encourage including these in the Reference list (authors, title, publisher (repository name), identifier, year).  For clinical datasets or third party data, please ensure that the statement adheres to our policy (https://www.nature.com/nature-research/editorial-policies/reporting-standards#availability-of-data) | The data availability statement has been included. And the data of our work can be shared upon request. |
| Please use the following template to provide all the information stated above:  The XX data generated in this study have been deposited in the YY database under accession code ZZ [add hyperlink here]. The XX data are available under restricted access for {insert reason}, access can be obtained by {explain how}. The raw XX data are protected and are not available due to data privacy laws. The processed XX data are available at YY. The XX data generated in this study are provided in the Supplementary Information/Source Data file. The XX data used in this study are available in the YY database under accession code ZZ [Add hyperlink here]. | The data are available under restricted access for academic request, access can be obtained by contacting the corresponding authors. |
| Specific advice on your Data Availability Statement: |  |
| Please consider making this data available in a publicly accessible repository, or explain why the data can only be made available from the authors on request. | The data availability statement has been included. And the data that support the findings of this study are available from the corresponding author upon reasonable request. Since all the necessary parameters have been put down in the manuscript and the numerical packages are easy to access by the readers, we choose to share our raw data upon request. |
|  |  |
|  |  |
| References |  |
| Our guidance: | Your response: |
| Supplementary References should appear at the end of the Supplementary Information file, and must be self-contained and numbered from 1. References mentioned in both the main text and the Supplementary Information should be part of both reference lists so that the Supplementary Information does not refer to the reference list in the main paper and vice versa. | The references in both the Supporting Information and the main text have been checked. |
|  |  |
| End matter |  |
| Our guidance: | Your response: |
| Nature Portfolio defines Competing Interest (CI) as financial and non-financial interests (including but not limited to funding, employment, stocks, shares, patents, personal or professional relationships with individuals or institutions, and unpaid membership advocacy) that could be perceived to directly undermine the objectivity, integrity, and value of a publication, or could be seen as having an influence on the judgments and actions of authors with regard to objective data presentation, analysis, and interpretation.  Please thoroughly review our policy on Competing Interests and include a detailed statement both in your final manuscript file and in our manuscript tracking system. Please ensure the statements are identical in both. Be specific about how each point stated relates to the research and list applicable author initials, and/or patent numbers.  If there are no competing interests, a negative statement must be included. | The authors declare no competing interests. And the statement has been included in the manuscript. |
| <https://www.nature.com/nature-research/editorial-policies/competing-interests> |  |
| Please confirm that all relevant funding awarded to each author is described in the Acknowledgements section. List each grant number, followed by the initials of the author who received it. | All funding with grant number, which is followed by the initials of the author who received it, has been described in the Acknowledgements section. |
|  |  |
| Preparing your manuscript files |  |
| Our guidance: | Your response: |
| Unless otherwise stated please limit individual file sizes to approximately 30MB. We strongly encourage the use of repositories for large datasets or source data due to size considerations. | Our individual file sizes have been limited to 30MB. |
| To ensure maximum visibility for your work, we may tweet about your paper following publication. If you would like us to include the Twitter handles of the first author(s), corresponding author(s), lab or institution in this tweet, please provide them in your cover letter. We would also welcome your suggestions for hashtags to use when tweeting about the work. | Please tweet our paper if possible. |
| Please supply the main manuscript file in either Microsoft Word or LaTeX format | The main manuscript file is in LaTex format. |
| Please provide figures as individual vector files with editable text. Acceptable file types for figures are .ai, .eps, .pdf or ChemDraw for fully editable vector-based art. For detailed guidance on figure preparation, see https://www.nature.com/documents/aj-artworkguidelines.pdf | The figures are vector files in pdf format. |
| The use or adaptation of previously published images is strongly discouraged. If this is unavoidable, please request the necessary rights documentation to re-use such material from the relevant copyright holders and return this to us when you submit your revised manuscript. Please check whether your manuscript or Supplementary Information contain third-party images, such as figures from the literature, stock photos, clip art or commercial satellite and map data.  For more information on what constitutes ownership by a third party, please contact our Editorial Assistant at naturecommunications@nature.com | We do not have previously published images in our paper. |
|  |  |
|  |  |
| Forms to complete |  |
| Our guidance: | Your response: |
| **Editorial Policy Checklist** |  |
| Please update and upload a final version of the Editorial Policy Checklist with your revised manuscript files. A blank Editorial Policy Checklist can be found via the link below. Note that this form is a dynamic ‘smart pdf’ and must be downloaded and completed in Adobe Reader. | We have uploaded the Editorial Policy Checklist. |
| Please update your current checklist or download from: |  |
| <https://www.nature.com/documents/nr-editorial-policy-checklist.zip> |  |
|  |  |
|  |  |
| **You will need to upload:** |  |
| Editorial Policy Checklist | We will upload Editorial Policy Checklist. |
| Completed Third Party Rights Table (if relevant) | Not applicable. |
| A point-by-point response to the reviewers' comments | We will upload the response. |
| A completed copy of this checklist | We will upload the checklist. |
| The main article file in Microsoft Word format - please supply a version with tracked changes and a version with tracked changes accepted | Our paper is in Latex format. |
| Separate Figure files | We will upload the figures. |
| Inventory of Supporting Information | The Supplementary Information includes a Supplementary Note pdf file only. |
| A Supplementary Information file | We will upload the Supplementary Information file. |
|  |  |
